# The Regional Specific Alterations in BBB Permeability are Relevant to the Differential Responses of 67-kDa LR Expression in Endothelial Cells and Astrocytes Following Status Epilepticus

**DOI:** 10.3390/ijms20236025

**Published:** 2019-11-29

**Authors:** Hana Park, Tae-Cheon Kang

**Affiliations:** 1 Department of Anatomy and Neurobiology, College of Medicine, Hallym University, Chuncheon 24252, Korea; M19050@hallym.ac.kr; 2 Institute of Epilepsy Research, College of Medicine, Hallym University, Chuncheon 24252, Korea

**Keywords:** AQP4, dystrophin, ERK1/2, hippocampus, JNK, PEA15, piriform cortex, SMI-71, U0126, vasogenic edema

## Abstract

Status epilepticus (a prolonged seizure activity, SE) differently affects vasogenic edema formation and dystrophin-aquaporin 4 (AQP4) expressions between the rat hippocampus and the piriform cortex (PC). In the present study, we explored whether the 67-kDa laminin receptor (LR) expression was relevant to the regional specific susceptibility of vasogenic edema at 3 days after SE. In spite of no difference in expression levels of 67-kDa LR, dystrophin, and AQP4 under physiological conditions, SE-induced serum extravasation was more severe in the PC than the hippocampus. Western blots demonstrated that SE reduced expression levels of 67-kDa LR, dystrophin, and AQP4 in the PC, but not in the hippocampus proper. Immunofluorescent studies revealed that SE increased 67-kDa LR expression in reactive CA1 astrocyte, but reduced it in the PC and the molecular layer of the dentate gyrus due to massive astroglial loss. Furthermore, SE decreased expressions of endothelial 67-kDa LR and SMI-71 (endothelial brain barrier antigen) in these regions. The 67-kDa LR neutralization evoked serum extravasation in these regions of normal animals without astroglial loss. Similar to SE, 67-kDa LR neutralization also reduced dystrophin-AQP4 expressions in the PC more than the total hippocampus. Furthermore, 67-kDa LR IgG infusion increased phosphorylation of extracellular signal-regulated kinase 1/2 (ERK1/2), but not c-Jun N-terminal kinase, independent of phosphoprotein enriched in astrocytes of 15 kDa (PEA15) activity. Co-treatment of U0126 (an ERK1/2 inhibitor) alleviated vasogenic edema formation and the reduced dystrophin-AQP4 expressions induced by 67-kDa LR neutralization. The 67-kDa LR IgG infusion also increased the susceptibility to SE induction. Therefore, our findings suggested that the cellular specific alterations in 67-kDa LR expression might be involved in the severity of SE-induced vasogenic edema formation in regional specific manners, which might affect the susceptibility to SE induction.

## 1. Introduction

Status epilepticus (a prolonged seizure activity, SE) is a neurologic emergency and one of the risk factors of developing acquired epilepsy [1,2]. In addition to neuronal death, SE evokes severe vasogenic edema in the hippocampus and the extrahippocampal limbic systems, including the piriform cortex (PC), due to the blood-brain barrier (BBB) disruption [3,4]. Furthermore, vasogenic edema up-regulates multidrug efflux transporter expressions, which become uncontrolled by conventional antiepileptic drugs (refractory seizure activity) [5]. Thus, vasogenic edema is considered as one of the modulating factors for pharmacoresistant temporal lobe epilepsy [6].

The BBB consists of endothelial cell, astrocyte, and gliovascular basement membrane (BM). Laminin is a major constituent of the BM of the BBB, which plays an important role in vascular development, vessel dilation, and physical integrity [7,8]. The laminin functionality is meditated by two cell-surface receptor families: the integrins and the non-integrin laminin receptors. The 67-kDa laminin receptor (67-kDa LR) is the first non-integrin laminin receptor identified by binding to immobilized laminin-1. The 67-kDa LR arises from a 32–33 kDa precursor, called p40 that is confirmed as a component of the 40S ribosome (ribosomal protein SA, RPSA) [9,10,11,12]. The 67-kDa LR plays a role in cell adherence to laminin and stabilizes or modulates the binding of laminin to other receptors [13,14]. Interestingly, blockade of 67-kDa LR evokes vasogenic edema by activating p38 mitogen-activated protein kinase (p38 MAPK)/vascular endothelial growth factor (VEGF), concomitant with decreasing the expressions of dystrophin-aquaporin 4 (AQP4) complex in the rat piriform cortex (PC) [15]. Since SE differently affects vasogenic edema formation between the hippocampus and the PC [3,4], it is likely that the distinct responses of 67-kDa LR expression would be relevant to the regional specific vasogenic formation in both regions following SE. 

On the other hand, 67-kDa LR regulates extracellular signal-regulated kinase 1/2 (ERK1/2), c-Jun N-terminal kinase (JNK), and p38 MAPK signaling pathways [16]. The 67-kDa LR activation also leads to the phosphorylation of phosphoprotein enriched in astrocytes of 15 kDa (PEA15) [17] that modulates various cellular functions [18]. Phosphorylations of serine (S) 104 and S116 sites of PEA15 increase ERK1/2 activity by inhibiting the binding of PEA15 to ERK1/2 [19,20,21,22,23]. With respect to these reports, elucidating the downstream effectors might be also noteworthy to understand the role of 67-kDa LR in vasogenic edema formation, albeit to a lesser extent.

Here, we demonstrated that SE reduced 67-kDa LR, dystrophin, and AQP4 expressions in the PC, but not in the total hippocampus, in spite of no difference in their expression levels in both regions under physiological conditions. Furthermore, the cellular specific alteration in 67-kDa LR expression affected the severity of SE-induced vasogenic edema formation in regional specific manners. Similar to SE, 67-kDa LR neutralization evoked serum extravasation in these regions of normal animals without astroglial loss. The 67-kDa LR neutralization also reduced dystrophin-AQP4 expressions in the PC more than the total hippocampus, independent of PEA15 activity. Furthermore, 67-kDa LR IgG infusion increased the susceptibility to SE induction. Therefore, our findings suggested that 67-kDa LR expression level might be involved in BBB integrity in regional specific manners, which might affect the susceptibility to SE induction.

## 2. Results

### 2.1. SE Differently Affects Expression Levels of 67-kDa LR, Dystrophin, and AQP4 in the Hippocampus and the PC

In a previous study [3], vasogenic edema peaked at 3 days after SE. Thus, we chose 3 days after SE as the best time point to explore the role of 67-kDa LR in vasogenic edema for western blot and immunohistochemistry. Although 67-kDa LR is expressed in neurons [24], we found that 67-kDa LR in astrocytes modulated AQP4 expression [15]. Therefore, we focused on the 67-kDa LR in astrocytes and endothelial cells in the present study. 

**Figure 1:**
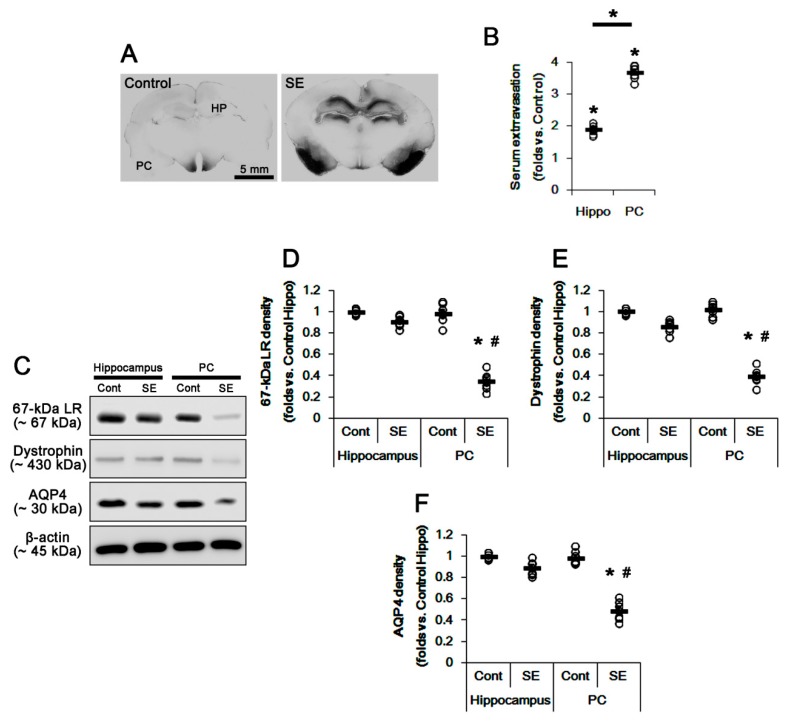
Vasogenic edema formation and expressions of 67-kDa LR, dystrophin, and AQP4 in the hippocampus and the PC at 3 days after SE. SE led to serum extravasation in the PC more than the hippocampus. In addition, expressions of 67-kDa LR, dystrophin, and AQP4 were decreased in the PC, but not in the hippocampus, at 3 days after SE. (**A**) Representative photographs for vasogenic edema in the hippocampus and the PC using immunohistochemistry for anti-rat IgG. (**B**) Quantitative values (mean ± S.E.M) of the serum extravasation in the hippocampus and the PC at 3 days after SE (*n* = 7, respectively). Open circles indicate each value. Horizontal bars indicate the mean value. Significant differences are * *p* < 0.05 vs. control animals and hippocampus (unpaired and paired Student’s *t*-test). (**C**) Representative western blot images for 67-kDa LR, dystrophin, and AQP4 in the hippocampus and the PC. (**D**–**F**) Quantitative values (mean ± S.E.M) of the western blot data concerning expression levels of 67-kDa LR (**D**), dystrophin (**E**), and AQP4 (**F**) at 3 days after SE (*n* = 7, respectively). Open circles indicate each value. Horizontal bars indicate the mean value. Significant differences are *^,#^ *p* < 0.05 vs. control animals and hippocampus (two-way ANOVA followed by Newman–Keuls posthoc test). LR: laminin receptor, AQP4: aquaporin 4, SE: status epilepticus, PC: piriform cortex.

Consistent with our previous studies [3,4,15], vasogenic edema was detected in the hippocampus (particularly in the CA1 region) and the PC (*p* < 0.05; unpaired Student’s *t*-test, *n* = 7, respectively; Figure 1A,B) at 3 days after SE. SE-induced serum extravasation (vasogenic edema) was more severe in the PC than the hippocampus (*p* < 0.05; paired Student’s *t*-test, *n* = 7, respectively; Figure 1A,B). In control animals, there was no difference in the expression levels of 67-kDa LR, dystrophin, and AQP4 between the total hippocampus and the PC (Figure 1C–F and Supplementary Figure S1). Three days after SE, the alterations in 67-kDa LR, dystrophin, and AQP4 expression levels were negligible in the total hippocampus (Figure 1C–F and Supplementary Figure S1). However, SE significantly reduced expression levels of 67-kDa LR, dystrophin, and AQP4 in the PC (*p* < 0.05 vs. control animals and hippocampus, two-way analysis of variance (ANOVA) followed by Newman–Keuls posthoc test, *n* = 7, respectively; Figure 1C–F and Supplementary Figure S1). 

**Figure 2:**
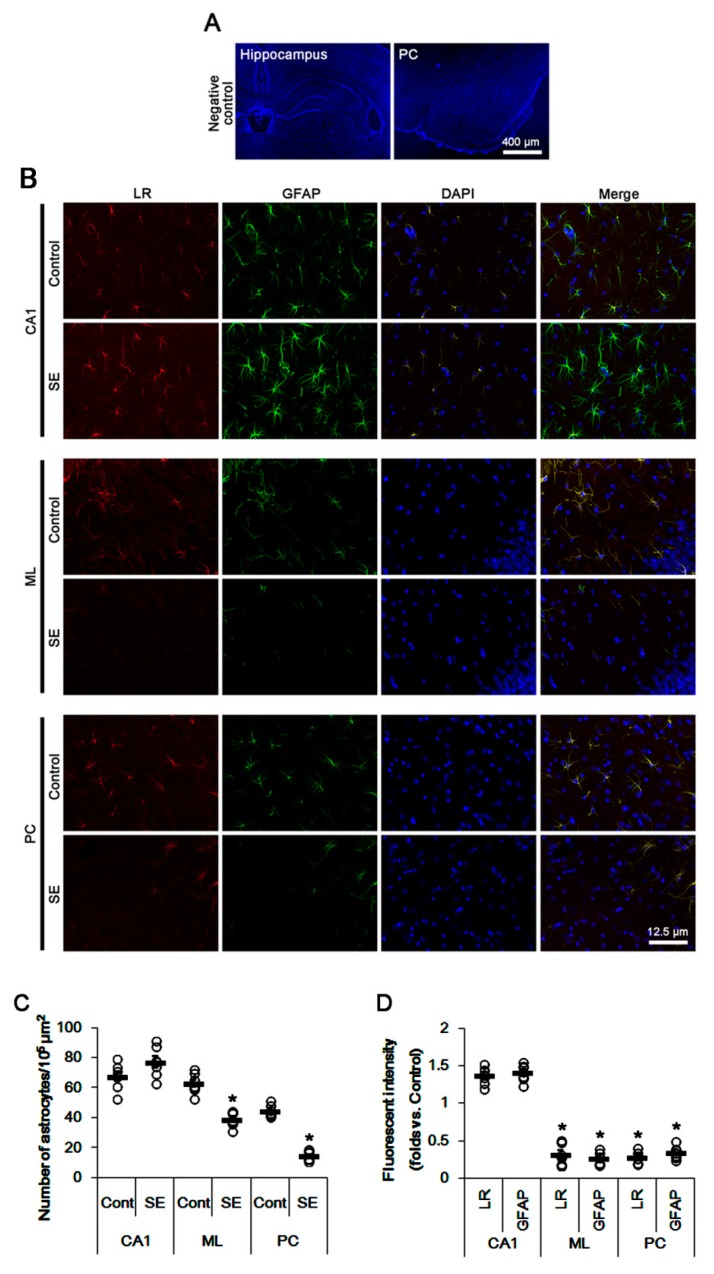
Expressions of 67-kDa LR (LR) and glial fibrillary acidic protein (GFAP, an astroglial maker) in the hippocampus and the PC at 3 days after SE. Reactive astrocytes showed strong 67-kDa LR expression in the CA1 region at 3 days after SE. However, 67-kDa LR expression was reduced in the molecular layer of the dentate gyrus (ML) and the PC due to SE-induced astroglial degenerations. (**A**) Representative photographs of negative control of 67-kDa LR in the hippocampus and the PC (DAPI counterstaining). (**B**) Representative photographs of 67-kDa LR and GFAP in the hippocampus and the PC. (**C**,**D**) Quantitative values (mean ± S.E.M) of the number of astrocytes (**C**) and the fluorescent intensities of 67-kDa LR and GFAP (**D**) at 3 days after SE (*n* = 7, respectively). Open circles indicate each value. Horizontal bars indicate the mean value. Significant differences are * *p* < 0.05 vs. control animals (unpaired Student’s *t*-test).

**Figure 3:**
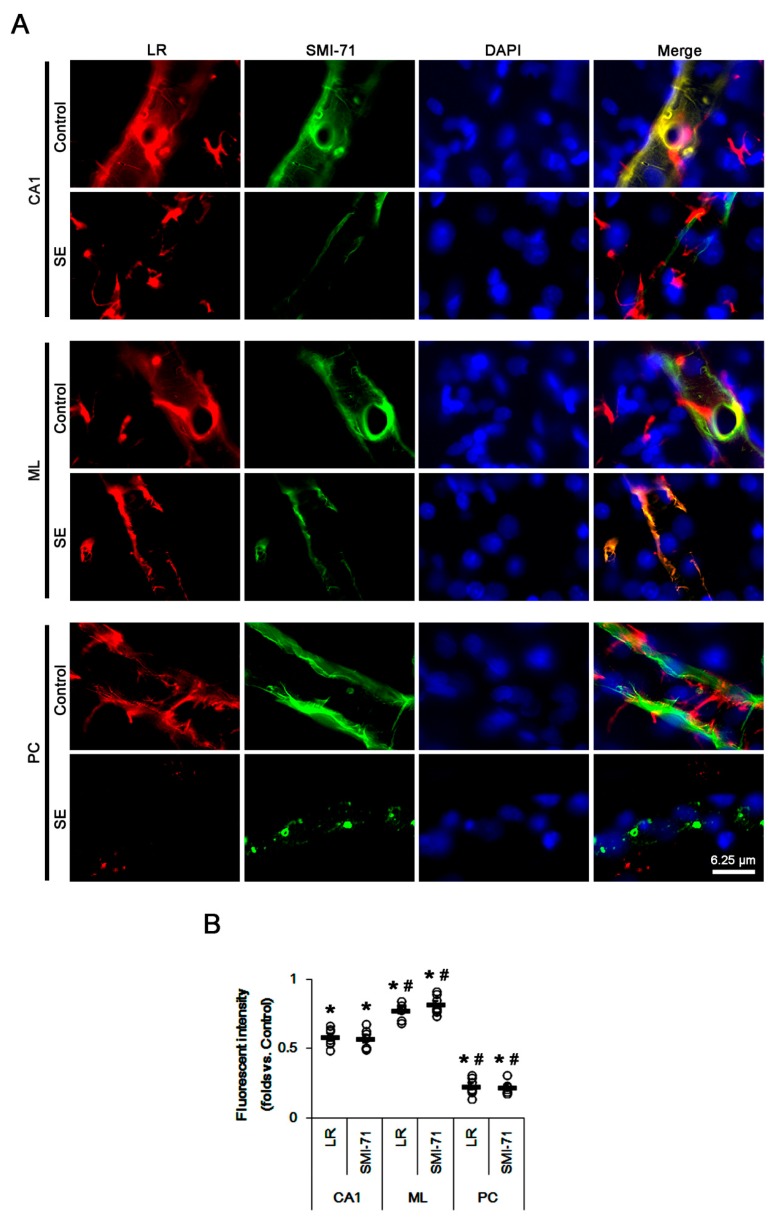
Expressions of 67-kDa LR (LR) and SMI-71 in the hippocampus and the PC at 3 days after SE. SE-induced down-regulations of 67-kDa LR and SMI-71 were detected in the PC more than the CA1 region and the molecular layer of the dentate gyrus (ML). In addition, 67-kDa LR and SMI-71 expressions were preserved in the ML more than the CA1 region. (**A**) Representative photographs of 67-kDa LR and SMI-71 in the hippocampus and the PC. (**B**) Quantitative values (mean ± S.E.M) of the fluorescent intensities of 67-kDa LR and SMI-71 at 3 days after SE (*n* = 7, respectively). Open circles indicate each value. Horizontal bars indicate the mean value. Significant differences are *^,#^ *p* < 0.05 vs. control animals (unpaired Student’s *t*-test) and CA1 region (one-way ANOVA followed by Newman–Keuls posthoc test), respectively.

Immunofluorescent studies revealed that astrocytes in the CA1 region showed reactive astrogliosis with increasing 67-kDa LR expression at 3 days after SE (*p* < 0.05 vs. control animals; unpaired Student’s *t*-test, *n* = 7, respectively; Figure 2A–D). In contrast, SE led to a massive astroglial loss and the reduced 67-kDa LR expression in the molecular layer of the dentate gyrus (ML) and the PC (*p* < 0.05 vs. control animals; unpaired Student’s *t*-test, *n* = 7, respectively; Figure 2B–D). Remaining astrocytes in the PC and the ML showed 67-kDa LR expression (Figure 2B). Furthermore, endothelial 67-kDa LR expression was decreased in these regions, concomitant with the decreased SMI-71 (endothelial brain barrier antigen, EBA) expression (*p* < 0.05 vs. control animals; unpaired Student’s *t*-test, *n* = 7, respectively; Figure 3A,B). However, the degree of these reductions was the PC > CA1 > ML (*p* < 0.05, one-way ANOVA followed by Newman–Keuls posthoc test, *n* = 7, respectively; Figure 3A,B). Therefore, our findings suggested that the alterations in 67-kDa LR expression between the hippocampus and the PC might be relevant to the astroglial viability and endothelial integrity, which would affect the differences of the severity of vasogenic edema formation and the reduction in dystrophin/AQP4 expressions following SE.

### 2.2. The 67-kDa LR Neutralization Decreases Dystrophin-AQP4 Expressions in the PC and the Hippocampus

**Figure 4:**
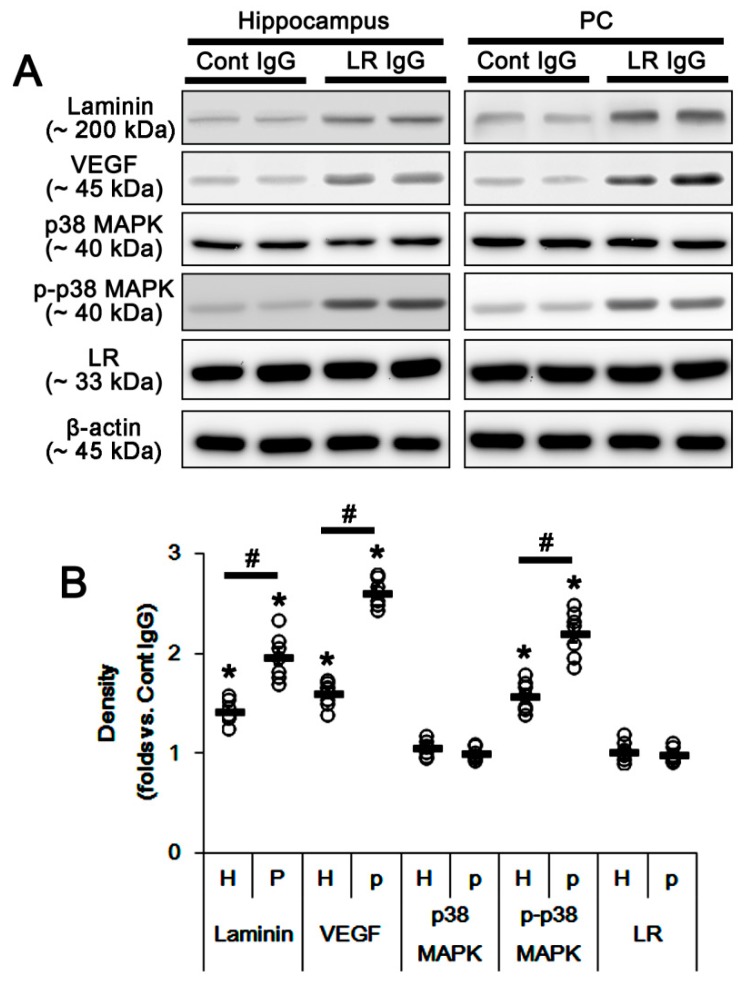
Effects of 67-kDa LR (LR) neutralization on expressions of laminin, VEGF, 67-kDa LR, and SMI-71, and p38 MAPK phosphorylation in the hippocampus and the PC of normal animals. The 67-kDa LR IgG infusion increased expressions of laminin and VEGF, and p38 MAPK phosphorylation in the PC more than the hippocampus without changing 67-kDa LR expression. The 67-kDa LR neutralization also diminished SMI-71 expression in the PC more than the hippocampus. (**A**) Western blot image for expression levels of laminin, VEGF, 67-kDa LR, and p38 MAPK phosphorylation induced by 67-kDa LR neutralization. (**B**) Quantitative values (mean ± S.E.M) of the western blot data concerning expression levels of laminin, VEGF, and 67-kDa LR, and p38 MAPK phosphorylation induced by 67-kDa LR neutralization (*n* = 7, respectively). Open circles indicate each value. Horizontal bars indicate the mean value. Significant differences are *^,#^ *p* < 0.05 vs. control IgG (unpaired Student’s *t*-test) and the hippocampus (paired Student’s *t*-test), respectively.

**Figure 5:**
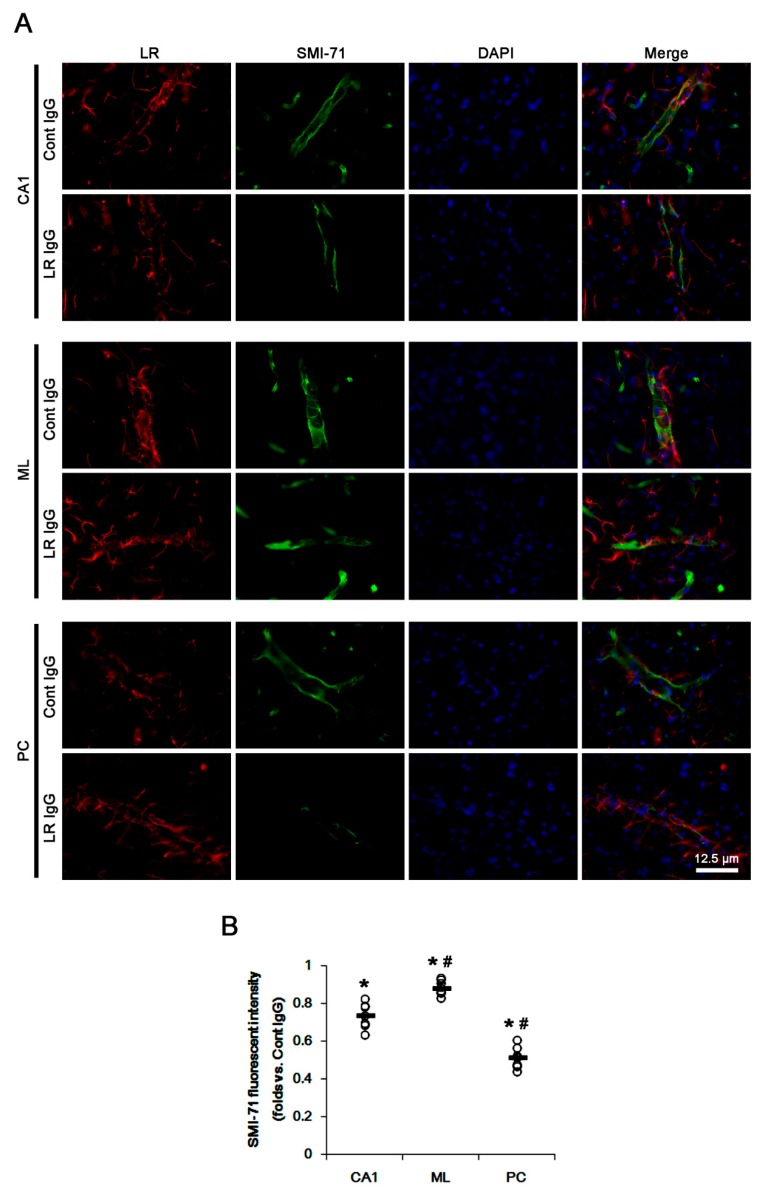
Effects of 67-kDa LR neutralization on SMI-71 expression in the hippocampus and the PC of normal animals. (**A**) Representative photographs of 67-kDa LR and SMI-71 in the hippocampus and the PC. (**B**) Quantitative values (mean ± S.E.M) of the fluorescent intensity of SMI-71 in the hippocampus and the PC (*n* = 7, respectively). Significant differences are *^,#^ *p* < 0.05 vs. control IgG (unpaired Student’s *t*-test) and CA1 region (one-way ANOVA followed by Newman–Keuls posthoc test), respectively.

Since SE evoked the massive astroglial loss in the present study, it was impossible to elucidate whether the down-regulation of dystrophin-AQP4 complex expression resulted from the reduced 67-kDa LR expression or astroglial degeneration induced by SE. To overcome these limitations, we applied 67-kDa LR neutralization to control animals. Laminin binding domain on 67-kDa LR is located at amino acid 209–229 regions. However, IgG binding to amino acid 272–280 regions of 67-kDa LR proves to be effective in the prevention of metastasis and angiogenesis while inducing apoptosis in cancers [25]. Since 67-kDa LR antibody used in the present study recognizes the amino acid 250–350 regions on 67-kDa LR, we had believed that 67-kDa LR neutralization would inhibit its downstream signaling cascades, independent of laminin-binding. Consistent with our previous study [15], Figure 4A shows 67-kDa LR IgG infusion increased expressions of laminin and VEGF, and p38 MAPK phosphorylation in the total hippocampus and the PC without changing 67-kDa LR expression level (*p* < 0.05 vs. control IgG, unpaired Student’s *t*-test, *n* = 7, respectively; Figure 4A,B and Supplementary Figure S2). The degree of these alterations was the PC > hippocampus (*p* < 0.05, paired Student’s *t*-test, *n* = 7, respectively; Figure 4A,B and Figure S2). The 67-kDa LR expression was detected in SMI-71-positive endothelial cells. The 67-kDa LR neutralization diminished SMI-71 expression in the parenchymal vessels in the hippocampus and the PC without altering 67-kDa LR expression (*p* < 0.05 vs. control IgG, unpaired Student’s *t*-test, *n* = 7, respectively; Figure 5A,B). These effects of 67-kDa LR neutralization were the PC > CA1 > ML (*p* < 0.05 vs. CA1, one-way ANOVA followed by Newman–Keuls posthoc test, *n* = 7, respectively; Figure 5A,B). These findings indicated that endothelial 67-kDa LR inhibition might increase BBB permeability under physiological conditions. 

**Figure 6:**
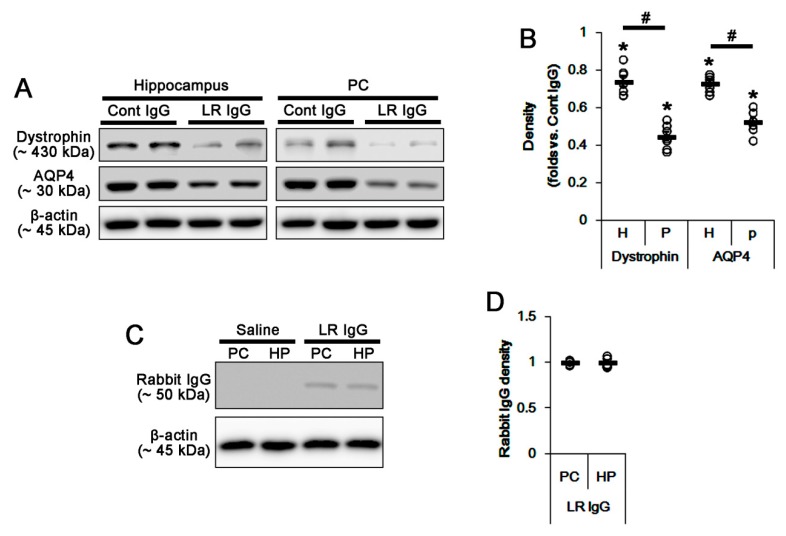
Effects of 67-kDa LR (LR) neutralization on expressions of dystrophin and AQP4 in the hippocampus and the PC of normal animals. The 67-kDa LR IgG infusion reduced expressions of dystrophin and the AQP4 in the PC more than the hippocampus. In addition, 67-kDa LR IgG was evenly diffused in the PC and the hippocampus after infusion. (**A**) Western blot image for expression levels of dystrophin and AQP4 following 67-kDa LR neutralization. (**B**) Quantitative values (mean ± S.E.M) of the western blot data concerning expression levels of dystrophin and AQP4 following 67-kDa LR neutralization (*n* = 7, respectively). Open circles indicate each value. Horizontal bars indicate the mean value. Significant differences are *^,#^ *p* < 0.05 vs. control IgG (unpaired Student’s *t*-test) and hippocampus (paired Student’s *t*-test), respectively. (**C**) Western blot image for rabbit IgG bands of 67-kDa LR antibody. (**D**) Quantitative values (mean ± S.E.M) of the western blot data concerning rabbit IgG bands following 67-kDa LR IgG infusion (*n* = 7, respectively). Open circles indicate each value. Horizontal bars indicate the mean value.

Next, we explored the effect of 67-kDa LR neutralization on dystrophin-AQP4 expressions, since dystrophin-AQP4 complex in astrocytes plays an important role in BBB integrity and serum extravasation [26,27,28]. The 67-kDa LR IgG infusion diminished dystrophin and AQP4 expressions in the total hippocampus and the PC (*p* < 0.05 vs. control IgG; unpaired Student’s *t*-test, *n* = 7, respectively; Figure 6A,B and Figure S3). The effects of 67-kDa LR neutralization on dystrophin-AQP4 expressions were more effective in the PC that those in the total hippocampus (*p* < 0.05; paired *t*-test, *n* = 7, respectively; Figure 6A,B and Figure S3). To confirm whether 67-kDa LR IgG evenly reaches the hippocampus and the PC after intracerebroventricular application, we quantified the amount of 67-kDa LR IgG remaining in the tissue by western blot using anti-rabbit IgG antibody. As a result, we found no difference in the amount of 67-kDa LR IgG between the PC and the total hippocampus (Figure 6C,D and Figure S3). Considering previous studies [3,29,30], these findings indicated the regional specific heterogeneous astroglial properties in the brain, and that the degree of AQP4 down-regulation might also affect the severity of serum extravasation between the PC and the hippocampus. 

### 2.3. The 67-kDa LR Neutralization Increases ERK1/2, but Not JNK, Phosphorylation

**Figure 7:**
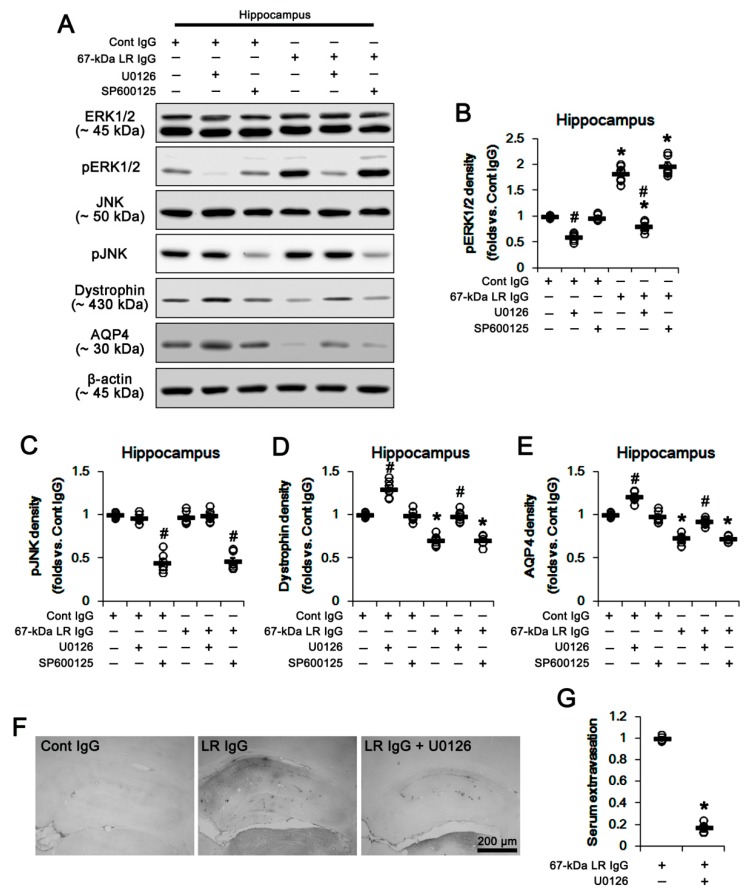
Effects of 67-kDa LR (LR) neutralization on ERK1/2 and JNK activities in the hippocampus of normal animals. U0126 (an ERK1/2 inhibitor), but not SP600125 (a JNK inhibitor), increased expressions of dystrophin and AQP4 in the hippocampus of control IgG-infused animals. The 67-kDa LR IgG infusion elevated pERK1/2, but reduced expressions of dystrophin and AQP4 in the hippocampus, which were attenuated by U0126. SP600125 (a JNK inhibitor) decreased only JNK phosphorylation. U0126 also mitigated vasogenic edema induced by 67-kDa LR neutralization. (**A**) Western blot image for expression and phosphorylation levels of ERK1/2, JNK, dystrophin, and AQP4. (**B**–**E**) Quantitative values (mean ± S.E.M) of the western blot data concerning phosphorylation and expression levels of ERK1/2 (**B**), JNK (**C**), dystrophin (**D**), and AQP4 (**E**) (*n* = 7, respectively). Open circles indicate each value. Horizontal bars indicate the mean value. Significant differences are *^,#^ *p* < 0.05 vs. control IgG and vehicle, respectively (one-way ANOVA followed by Newman–Keuls posthoc test). (**F**) Representative photographs for serum extravasation in the hippocampus induced by 67-kDa LR neutralization. (**G**) Quantitative values (mean ± S.E.M) of the serum extravasation in the hippocampus (*n* = 7, respectively). Open circles indicate each value. Horizontal bars indicate the mean value. Significant differences are * *p* < 0.05 vs. 67-kDa LR IgG (unpaired Student’s *t*-test). ERK1/2: extracellular signal-regulated kinase 1/2, JNK: c-Jun N-terminal kinase.

Because 67-kDa LR regulates ERK1/2 and JNK activities [16], we investigated whether blockade of 67-kDa LR influences these kinase activities (phosphorylations) in both regions. As compared to control IgG, 67-kDa LR IgG infusion significantly increased ERK1/2 phosphorylation without changing ERK1/2 expression level in the total hippocampus, while it did not affect JNK expression and its phosphorylation (*p* < 0.05 vs. control IgG, respectively; one-way ANOVA followed by Newman–Keuls posthoc test, *n* = 7, respectively; Figure 7A–C and Figure S4). Since 67-kDa LR neutralization effectively increased ERK1/2 phosphorylation in the total hippocampus, we applied co-treatment of U0126 (an ERK1/2 inhibitor) or SP600125 (a JNK inhibitor) to validate the role of ERK1/2 and JNK activities in the reduced dystrophin-AQP4 expressions induced by 67-kDa LR neutralization. Co-treatment of U0126 with control IgG significantly increased dystrophin-AQP4 expressions in the total hippocampus, but reduced ERK1/2 phosphorylation (*p* < 0.05 vs. vehicle; one-way ANOVA followed by Newman–Keuls posthoc test, *n* = 7, respectively; Figure 7A,B,D,E and Figure S4). In addition, U0126 co-treatment alleviated the decreases in dystrophin-AQP4 expressions induced by 67-kDa LR neutralization, accompanied by decreasing ERK1/2 phosphorylation (*p* < 0.05 vs. vehicle; one-way ANOVA followed by Newman–Keuls posthoc test, *n* = 7, respectively; Figure 7A,B,D,E and Figure S4). However, co-treatment of SP600125 did not affect dystrophin-AQP4 expressions and ERK1/2 phosphorylation in the total hippocampus of control IgG- and 67-kDa LR IgG-infused animals, while it diminished JNK phosphorylation (*p* < 0.05 vs. vehicle; one-way ANOVA followed by Newman–Keuls posthoc test, *n* = 7, respectively; Figure 7A,C and Figure S4). Furthermore, U0126 effectively inhibited serum extravasation in the hippocampus induced by 67-kDa LR neutralization (*p* < 0.05 vs. vehicle; unpaired Student’s *t*-test, *n* = 7, respectively; Figure 7F,G). 

**Figure 8:**
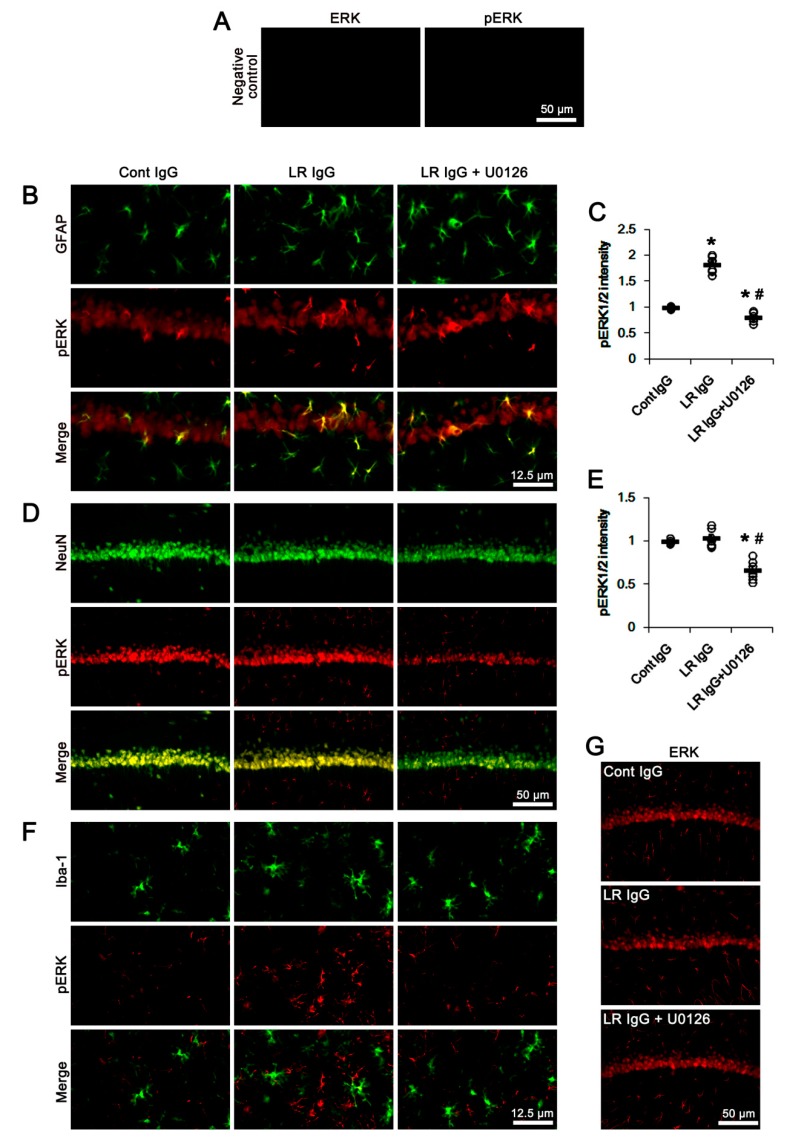
Effects of 67-kDa LR (LR) neutralization on ERK1/2 and pERK1/2 levels in the hippocampus of normal animals. The 67-kDa LR IgG infusion elevated pERK1/2 level in astrocytes, but not CA1 neurons and microglia, without altering ERK1/2 expression. U0126 attenuated pERK1/2, but not EKR, level in these cells following 67-kDa LR IgG infusion. (**A**) Representative photographs of negative control of ERK1/2 and p-ERK1/2 antibodies. (**B**) Representative photographs for pERK1/2 level in hippocampal astrocytes. (**C**) Quantitative values (mean ± S.E.M) of pERK1/2 level in hippocampal astrocytes (*n* = 7, respectively). Open circles indicate each value. Horizontal bars indicate the mean value. Significant differences are *,^#^ *p* < 0.05 vs. control IgG and 67-kDa LR IgG, respectively (one-way ANOVA followed by Newman–Keuls posthoc test). (**D**) Representative photographs for pERK1/2 level in CA1 neurons (NeuN, a neuronal marker). (**E**) Quantitative values (mean ± S.E.M) of pERK1/2 level in CA1 neurons (*n* = 7, respectively). Open circles indicate each value. Horizontal bars indicate the mean value. Significant differences are *^,#^ *p* < 0.05 vs. control IgG and 67-kDa LR IgG, respectively (one-way ANOVA followed by Newman–Keuls posthoc test). (**F**) Representative photographs for pERK1/2 level in hippocampal microglia (Iba-1, a microglia marker). (**G**) Representative photographs for ERK1/2 level in the hippocampal CA1 region.

**Figure 9:**
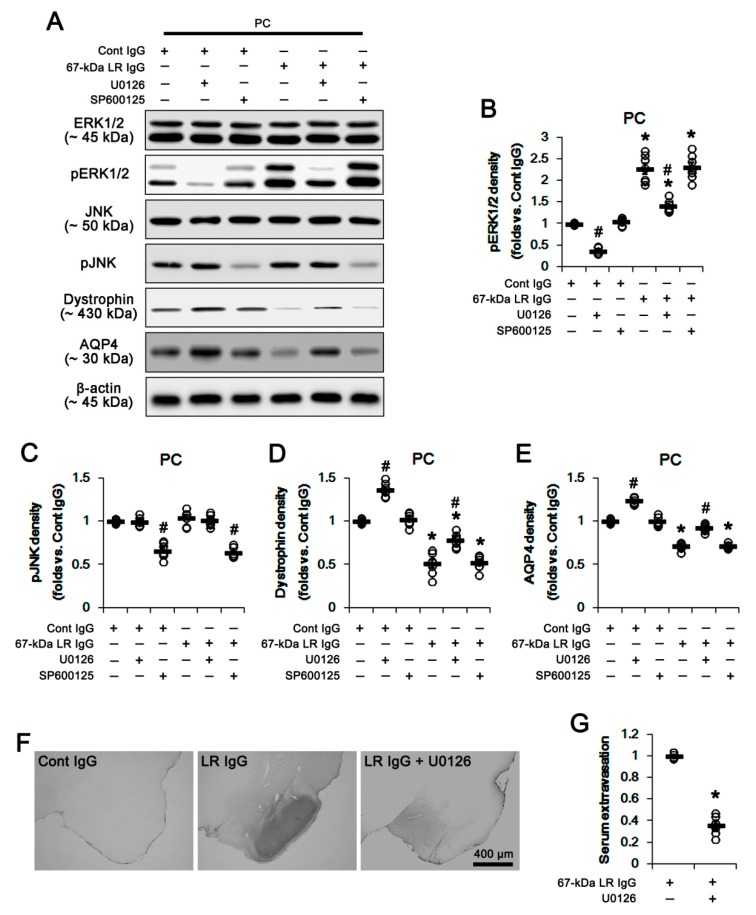
Effects of 67-kDa LR (LR) neutralization on ERK1/2 and JNK activities in the PC of normal animals. U0126 (an ERK1/2 inhibitor), but not SP600125 (a JNK inhibitor), increased expressions of dystrophin and AQP4 in the PC of control IgG-infused animals. The 67-kDa LR IgG infusion elevated pERK1/2, but reduced expressions of dystrophin and AQP4 in the PC, which were attenuated by U0126. SP600125 (a JNK inhibitor) decreased only JNK phosphorylation. U0126 also mitigated vasogenic edema induced by 67-kDa LR neutralization. (**A**) Western blot image for expression and phosphorylation levels of ERK1/2, JNK, dystrophin, and AQP4. (**B**–**E**) Quantitative values (mean ± S.E.M) of the western blot data concerning phosphorylation and expression levels of ERK1/2 (**B**), JNK (**C**), dystrophin (**D**), and AQP4 (**E**) (*n* = 7, respectively). Open circles indicate each value. Horizontal bars indicate the mean value. Significant differences are *^,#^ *p* < 0.05 vs. control IgG and vehicle, respectively (one-way ANOVA followed by Newman–Keuls posthoc test). (**F**) Representative photographs for serum extravasation in the PC induced by 67-kDa LR neutralization. (**G**) Quantitative values (mean ± S.E.M) of the serum extravasation in the PC (*n* = 7, respectively). Open circles indicate each value. Horizontal bars indicate the mean value. Significant differences are * *p* < 0.05 vs. 67-kDa LR IgG (unpaired Student’s *t*-test).

**Figure 10:**
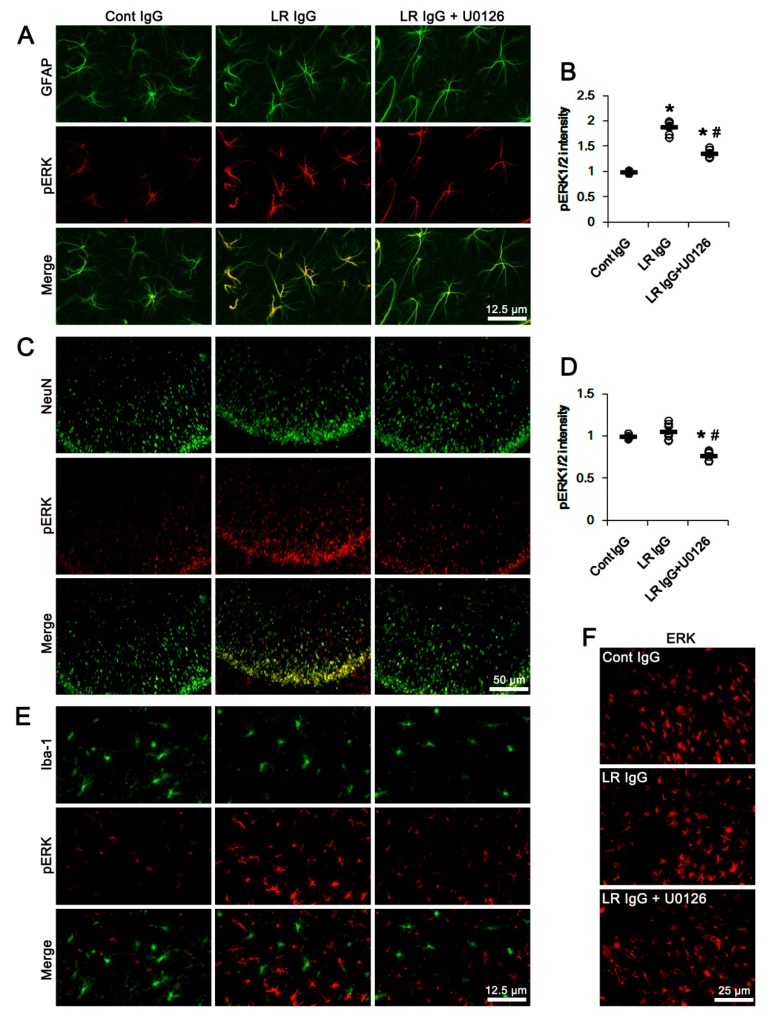
Effects of 67-kDa LR (LR) neutralization on ERK1/2 and pERK1/2 expression in the PC of normal animals. Similar to the hippocampus, 67-kDa LR IgG infusion elevated pERK1/2 level in astrocytes, but not CA1 neurons and microglia, without changing ERK1/2 expression. U0126 attenuated pERK1/2, but not ERK1/2, levels in these cells following 67-kDa LR IgG infusion. (**A**) Representative photographs for pERK1/2 level in the PC astrocytes. (**B**) Quantitative values (mean ± S.E.M) of pERK1/2 level in the PC astrocytes (*n* = 7, respectively). Open circles indicate each value. Horizontal bars indicate the mean value. Significant differences are *,^#^ *p* < 0.05 vs. control IgG and 67-kDa LR IgG, respectively (one-way ANOVA followed by Newman–Keuls posthoc test). (**C**) Representative photographs for pERK1/2 level in the PC neurons (NeuN, a neuronal marker). (**D**) Quantitative values (mean ± S.E.M) of pERK1/2 level in PC neurons (*n* = 7, respectively). Open circles indicate each value. Horizontal bars indicate the mean value. Significant differences are *^,#^ *p* < 0.05 vs. control IgG and 67-kDa LR IgG, respectively (one-way ANOVA followed by Newman–Keuls posthoc test). (**E**) Representative photographs for pERK1/2 level in the PC microglia (Iba-1, a microglia marker). (**F**) Representative photographs for ERK1/2 level in the PC.

The double immunofluorescent study revealed pERK1/2 signals were mainly observed in astrocytes and neurons. A few microglia showed pERK1/2 expression (Figure 8). The 67-kDa LR neutralization and co-treatment of U0126 affected ERK1/2 phosphorylation level in astrocytes without an astroglial loss (*p* < 0.05 vs. control IgG and 67-kDa LR IgG, respectively; one-way ANOVA followed by Newman–Keuls posthoc test, *n* = 7, respectively; Figure 8A–C). However, 67-kDa LR IgG did not affect ERK1/2 phosphorylation levels in neurons and microglia, although pERK1/2 levels in these cells were reduced by U0126 co-treatment (Figure 8D–F). The ERK1/2 expression level was unaffected by 67-kDa LR IgG and U0126 (Figure 8G). These effects of U0126 co-treatment on dystrophin-AQP4 expressions, ERK1/2 phosphorylation, and serum extravasation were similarly observed in the PC (*p* < 0.05 vs. vehicle; one-way ANOVA followed by Newman–Keuls posthoc test and unpaired Student’s *t*-test, *n* = 7, respectively; Figure 9A–G, Figure 10A–F and Figure S4). Taken together, our findings suggested that blockade of 67-kDa LR might diminish dystrophin-AQP4 expressions, but increase astroglial ERK1/2 activation in the hippocampus and the PC under physiological conditions.

### 2.4. The 67-kDa LR Neutralization Does Not Affect PEA15 Phosphorylations

**Figure 11:**
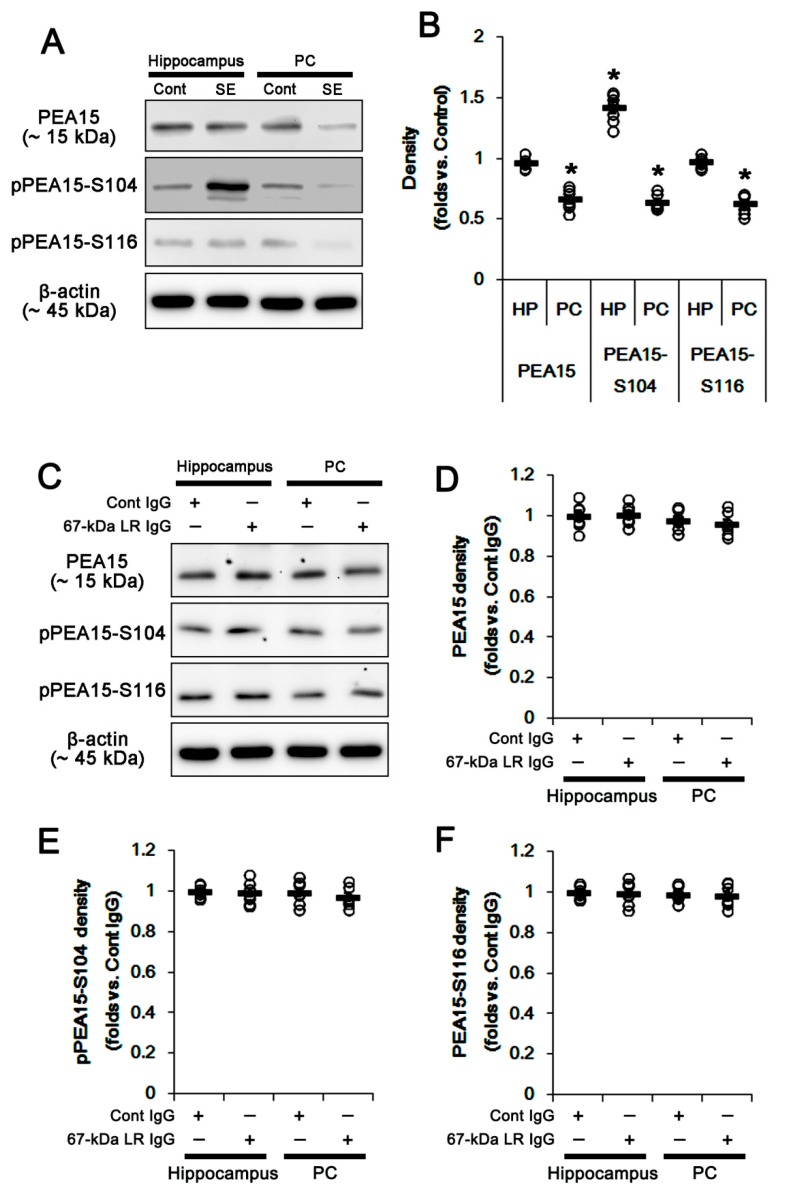
Effects of SE and 67-kDa LR (LR) neutralization on PEA15 phosphorylations in the hippocampus and the PC. PEA15-S104 phosphorylation was up-regulated in the hippocampus at 3 days after SE, although it did not affect PEA15 expression and its S116 phosphorylation. In contrast, SE decreased PEA15 expression and its phosphorylations in the PC (**A**,**B**). The 67-kDa LR IgG infusion did not affect PEA15 expression and its phosphorylations in the hippocampus and the PC (**C–F**). (**A**) Representative western blot images for PEA15 expression and its phosphorylation in the hippocampus and the PC at 3 days after SE. (**B**) Quantitative values (mean ± S.E.M) of the western blot data concerning PEA15 expression and its phosphorylation at 3 days after SE (*n* = 7, respectively). Open circles indicate each value. Horizontal bars indicate the mean value. Significant differences are * *p* < 0.05 vs. control animals (unpaired Student’s *t*-test). (**C**) Western blot image for expression and phosphorylation levels of PEA15 following control IgG and 67-kDa LR infusion. (**D**–**F**) Quantitative values (mean ± S.E.M) of the western blot data concerning PEA15 expression and its phosphorylation (*n* = 7, respectively). PEA15: phosphoprotein enriched in astrocytes of 15 kDa.

PEA15 is a small phosphoprotein, which is abundantly expressed in astrocytes [23]. The phosphorylation of PEA15 at S104 and/or S116 site increases ERK1/2 activity by abrogating PEA15-ERK1/2 interaction [23]. Thus, we explored the effects of SE and 67-kDa LR neutralization on PEA15 expression and its phosphorylations. PEA15-S104 phosphorylation was up-regulated in the total hippocampus at 3 days after SE, although PEA15 expression and its S116 phosphorylation were unaltered. In contrast, SE decreased PEA15 expression and its phosphorylations in the PC (*p* < 0.05 vs. control animals; unpaired Student’s *t*-test, *n* = 7, respectively; Figure 11A,B and Figure S5). The 67-kDa LR IgG infusion did not alter PEA15 expression and its phosphorylations in the total hippocampus and the PC (Figure 11C–F and Figure S5). Thus, our findings suggested that blockade of 67-kDa LR might not affect PEA15 phosphorylations under physiological- and post-SE conditions.

### 2.5. The 67-kDa LR Neutralization Increases the Susceptibility to SE Induction

**Figure 12:**
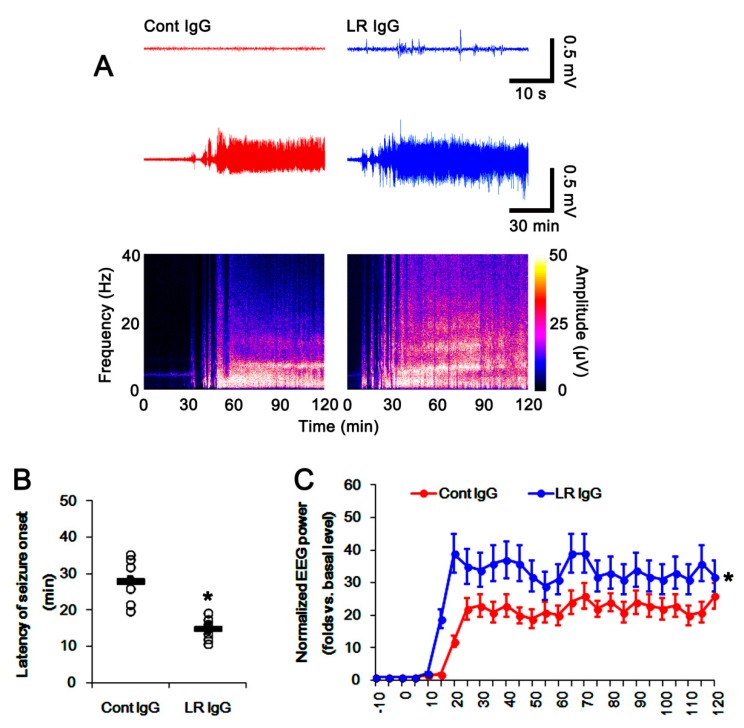
Effects of control IgG and 67-kDa LR IgG on seizure activity in response to pilocarpine. As compared to control IgG infusion, 67-kDa LR neutralization induced paroxysmal discharges on the baseline EEG in the hippocampus 3 days after infusion. In addition, 67-kDa LR IgG reduced seizure latency and increased seizure severity in response to pilocarpine. The 67-kDa LR IgG infusion reduced seizure latency and increased seizure severity in response to pilocarpine. (**A**) Representative baseline EEG (upper traces), seizure activity (lower traces), and frequency-power spectral temporal maps in response to pilocarpine. (**B**) Quantification of latency of seizure onset in response to pilocarpine (mean ± S.E.M.; *n* = 7, respectively). Open circles indicate each value. Horizontal bars indicate the mean value. Significant differences are * *p* < 0.05 vs. control IgG (unpaired Student’s *t*-test). (**C**) Quantification of total EEG power (seizure intensity) in response to pilocarpine (mean ± S.E.M.; *n* = 7, respectively). Significant differences are * *p* < 0.05 vs. control IgG (one-way repeated measure ANOVA). EEG: electroencephalogram.

Since leakage of serum-derived components into the extracellular space provokes neuronal hyperexcitability following vasogenic edema [31], it is likely that 67-kDa LR neutralization may affect susceptibility to SE induction. To confirm this, we evaluated the effects of 67-kDa LR IgG infusion on the latency of seizure onset and total EEG (electroencephalogram) power in response to pilocarpine. Although 67-kDa LR neutralization did not induce behavioral seizure activity, it evoked paroxysmal discharges on the baseline EEG in the hippocampus 3 days after infusion and increased the susceptibility to SE induction (*p* < 0.05 vs. control IgG; one-way repeated measure ANOVA, *n* = 7, respectively; Figure 12A–C). Therefore, these findings indicated that 67-kDa LR neutralization might influence the susceptibility to SE induction and seizure severity.

## 3. Discussion

The major findings in the present study were that SE resulted in the cellular specific alterations in 67-kDa LR expression, which was relevant to the different BBB permeability between the hippocampus and the PC. In addition, 67-kDa LR neutralization distinctly affected endothelial BBB integrity and dystrophin-AQP4 expression in both regions. These findings suggested that the altered 67-kDa LR functionality might be involved in the regional specific changes in vascular permeability following SE.

In the present study, there was no difference in expression levels of 67-kDa LR between the total hippocampus and the PC of control animals. Three days after SE, vasogenic edema formation was observed in the PC, accompanied by the reduced 67-kDa LR expression. SE also evoked serum extravasation in the hippocampus, particularly in the CA1 region, without altered total 67-kDa LR expression in western blots. However, immunohistochemical studies revealed the cellular specific responses of 67-kDa LR expression to SE in the hippocampus and the PC: In the CA1 region, 67-kDa LR expression was decreased in endothelial cells but increased in reactive astrocytes. In the ML, SE-induced astroglial loss reduced 67-kDa LR expression in astrocytes more than endothelial cells. In the PC, SE similarly diminished its expression in both astrocytes and endothelial cells. Thus, the degree of reductions of SMI-71 expression was the PC > CA1 > ML, which was similar to the severity of serum extravasation at 3 days after SE. Furthermore, 67-kDa LR neutralization reduced SMI-71 expression in the parenchymal vessels in the hippocampus and the PC without altering 67-kDa LR expression. The effect of 67-kDa LR neutralization on SMI-71 was the PC > CA1 > ML, which was similar to the increased vascular permeability. Therefore, our findings suggested that the altered 67-kDa LR functionality in endothelial cells might trigger serum extravasation, and be involved in the differential vascular permeability in the PC, CA1 region, and ML following SE. 

**Figure 13:**
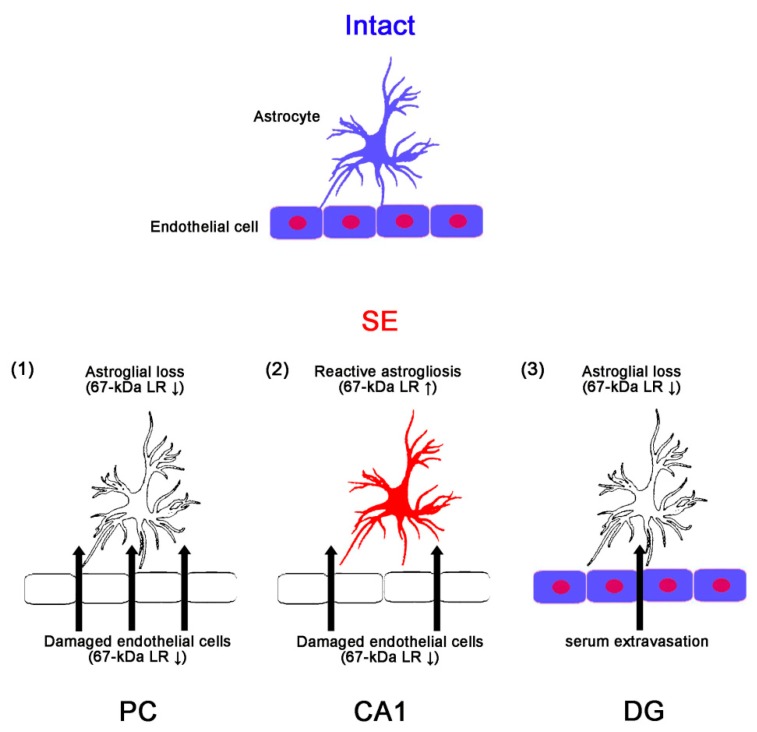
Scheme of the chronological alterations of 67-kDa LR expression in the PC, CA1, and the molecular layer of the dentate gyrus (DG) based on the present data and previous reports [3,4,29,42]. Under physiological conditions, 67-kDa LR is expressed in intact astrocytes and endothelial cells in all regions. (**1**) Twelve hours to three days after SE, 67-kDa LR expression is reduced in the PC due to astroglial loss and endothelial cell damage. (**2**) In addition, 67-kDa LR expression is declined only in the endothelial cells within the CA1 region at 1–3 days after SE. Therefore, vasogenic edema in the CA1 region is less severe than that in the PC. (**3**) Subsequently, 67-kDa LR expression is decreased in the DG at 3–7 days after SE because of massive astroglial loss. The maintenance of endothelial integrity minimizes serum extravasation. Thus, the cellular specific alteration in 67-kDa LR may be involved in the regional specific severity of vasogenic edema induced by SE.

Although AQP4 deletion cannot evoke serum extravasation, it deteriorates vasogenic edema formation. This is because vasogenic water elimination occurs through AQP4-dependent routes [32,33]. Indeed, the decreased dystrophin-AQP4 expressions are also involved in vasogenic edema formation in the PC following SE [3,4]. In addition, inhibition of AQP4 by acetazolamide (AZA) aggravates SE-induced vasogenic edema and astroglial loss in the PC, while it does not evoke vasogenic edema in control animals [3]. Deletion of dystrophin also reduces AQP4 expression over perivascular astroglial end foot membranes, which influences the degree of vasogenic edema formation [3,34,35,36,37,38]. In contrast to the PC, the hippocampus shows mild, not absent, changes in dystrophin-AQP4 expressions and serum-protein extravasations after SE [3]. In addition, AZA deteriorates vasogenic edema and astroglial loss in the hippocampus, particularly in the stratum radiatum of the CA1 region without affecting astroglial loss in the molecular layer of the dentate gyrus [3]. Thus, we had speculated these discrepancies would be due to regional-specific susceptibility to SE induction or the difference of anatomical characteristics between the hippocampus and the PC. Indeed, PC is highly sensitive to pilocarpine-induced SE rather than the hippocampus [39,40,41]. Furthermore, SE results in an acute and devastating astroglial loss, which is characterized by a pattern of selective vulnerability [29,42,43,44]. In the present study, SE led to an astroglial loss in the PC and the ML. Furthermore, remaining astrocytes showed 67-kDa LR expression in these regions. Thus, it is likely that SE-induced astroglial loss might result in the down-regulation of 67-kDa LR in astrocytes. In addition, it is plausible that SE-induced astroglial loss would evoke vasogenic edema, regardless of astroglial 67-kDa LR expression. In the present study, however, SE evoked serum extravasation in the CA1 region without astroglial loss, but not in the ML, where it showed astroglial degeneration. Indeed, vasogenic edema spreads from the CA1 regions to all hippocampal regions following SE [3]. Consistent with a previous study [15], the present study also revealed that 67-kDa LR neutralization evoked vasogenic edema formation in the PC than the total hippocampus without astroglial loss. Considering the SE-induced reduction in SMI-71 expression, these findings indicated that astroglial degeneration/dysfunction might not be essential for the initiation of vasogenic edema formation. On the other hand, the present data showed that astrocytes in the CA1 region showed reactive astrogliosis with increasing 67-kDa LR expression at 3 days after SE. In addition, the alterations in 67-kDa LR, dystrophin, and AQP4 expression levels were negligible in the total hippocampus, as compared to the PC. The 67-kDa LR neutralization also diminished the dystrophin-AQP4 complex within the PC than the total hippocampus. These findings indicated that astroglial 67-kDa LR might contribute to maintaining astroglial BBB integrity, which might facilitate the recovery of serum extravasation via dystrophin-AQP4 complex. Together with previous studies [3,4,29,42], the present data demonstrated the time-sequencing resume of events concerning 67-kDa LR-mediated serum extravasation as follows: Under physiological condition, 67-kDa LR was expressed in intact astrocytes and endothelial cells in all regions. Twelve hours to three days after SE, 67-kDa LR expression was reduced in the PC due to astroglial loss and endothelial cell damage. Subsequently, 67-kDa LR expression was declined only in the endothelial cells within the CA1 region at 1–3 days after SE. Therefore, vasogenic edema in the CA1 region was less severe than that in the PC. Finally, 67-kDa LR expression was decreased in the molecular layer of the dentate gyrus (DG) at 3–7 days after SE because of massive astroglial loss. The maintenance of endothelial integrity minimized serum extravasation (Figure 13). Therefore, our findings suggested that the maintenance of 67-kDa LR expression might be involved in the distinct severity of SE-induced vasogenic edema formation between the hippocampus and the PC by regulating dystrophin-AQP4 expression.

Givant-Horwitz et al. [16] reported that antisense-67-kDa LR expressing melanoma cells showed the higher basal ERK1/2 phosphorylation level than parental cells and sense-transfected cells. In addition, exogenous soluble laminin decreased ERK1/2 phosphorylation, irrespective of the 67-kDa LR expression level. Similar to exogenous soluble laminin, epigallocatechin-3-gallate (EGCG, a green tea polyphenol), known as another ligand of 67-kDa LR [45], inhibits ERK1/2 phosphorylation in activated macrophages and dendritic cells [46,47]. In addition, Ku and his colleagues demonstrated that EGCG inhibited the insulin- or insulin-like growth factor (IGF)-induced ERK1/2 phosphorylation in preadipocytes, which was abrogated by 67-kDa LR neutralization [48,49]. Thus, it is likely that 67-kDa LR might inhibit ERK1/2 phosphorylation. Consistent with these previous studies, the present study showed that 67-kDa LR neutralization increased ERK1/2 phosphorylation in the total hippocampus and the PC, accompanied by reducing dystrophin-AQP4 expressions. Furthermore, U0126 effectively abolished serum extravasation and down-regulation of dystrophin-AQP4 complex induced by 67-kDa LR neutralization. These findings indicated that the dysfunction of 67-kDa LR in intact astrocytes might diminish dystrophin-AQP4 complex via ERK1/2 activation. However, the role of ERK1/2 in AQP4 regulation has been still controversial. Shi et al. [50] reported that ERK1/2 activation decreased AQP4 expression in cultured astrocyte following scratch-injury, while Qi et al. [51] demonstrated that ERK1/2 enhanced AQP4 expression induced by oxygen-glucose deprivation. Salman et al. [52] also described that ERK1/2 activity did not affect AQP4 expression in primary human astrocytes. Furthermore, SE did not affect the hippocampal dystrophin-AQP4 expression in the present study, although it decreased ERK1/2 phosphorylation in the hippocampus due to neuronal death [53,54,55]. Regarding the signaling pathway concerning the regulation of dystrophin-AQP4 expression by U0126, one considerable possibility is that U0126 would directly influence 67-kDa LR expression. This is because U0126 inhibits hypoxia-induced 67-kDa LR expression in MKN-45 gastric cancer cells [56]. U0126 reduces 67-kDa LR expression; however, in the present study, co-treatment of U0126 would exacerbate serum extravasation and down-regulation of dystrophin-AQP4 expressions induced by 67-kDa LR IgG. Another possibility is that U0126 would affect dystroglycan-mediated AQP4 regulation. Dystroglycan forms a complex with dystrophin and anchors AQP4 on the cell membrane. Interestingly, dystroglycan increases AQP4 expression by ERK1/2 activation, which is blocked by U0126 [57]. However, the present study showed that U0126 increased dystrophin-AQP4 expression. Considering these previous studies and the present data, it is likely that ERK1/2 might not be directly involved in the regulation of dystrophin-AQP4 expression following SE, or that U0126 might modulate dystrophin-AQP4 expression via ERK1/2-mediated unknown pathways in intact astrocytes. Further studies are needed to elucidate signaling pathways involved in the regulation of AQP4 expression after 67-kDa LR neutralization. 

In neurons, 67-kDa LR interacts with cellular prion protein, which is involved in its internalization [58]. The 37-kDa precursor forms also bind to the growth factor midkine [59] and cytotoxic necrotizing factor 1, a bacterial toxin inducing host cell cytoskeleton rearrangements [60]. Interestingly, the 37-kDa precursor form is exclusively expressed in parvalbumin cells [24] that are rapidly degenerated induced by SE [61,62]. Furthermore, 67-kDa LR regulates neurite outgrowth activity [63]. However, there is a lack of evidence to support the role of 67-kDa LR in neuronal excitability or seizure activity. In the present study, we found that 67-kDa LR neutralization evoked paroxysmal discharges on the baseline EEG in the hippocampus 3 days after infusion and increased the susceptibility to SE induction. Regarding the role of serum extravasation in neuronal hyperexcitability [31], it is likely that 67-kDa LR neutralization may reduce seizure threshold in response to pilocarpine, induced by vasogenic edema. Furthermore, it is considered that 67-kDa LR IgG would enhance the penetration of pilocarpine across BBB. Since pilocarpine has a poor permeability across the intact BBB, high doses of pilocarpine are required for SE induction. Furthermore, pilocarpine alters BBB permeability before ictogenesis [64]. Indeed, vascular permeability affects the sensitivity of seizure activity to pilocarpine: LiCl decreases the concentration of pilocarpine for SE induction due to the increased BBB permeability [65]. Thus, it is likely that 67-kDa LR neutralization may facilitate the entrance of pilocarpine (reduced onset) and the increased SE severity (higher concentration of pilocarpine into the brain) by BBB alterations. Because both conditions (the serum extravasation and the increased pilocarpine penetration) are required for the increased vascular permeability [31,64,65], our findings suggested that 67-kDa LR functionality might be relevant to a different susceptibility of the PC and the hippocampus to SE generation via BBB integrity. In addition, the reduced AQP4 expression would be involved in the changed pilocarpine-induced seizure activity following 67-kDa LR IgG infusion. In spite of the absence of vasogenic edema, AQP4 deletion increases electrical stimulation-induced seizure duration in mice [66] and the frequency of seizures during the first week after kainic acid-induced SE [67]. Furthermore, deficiency of α-syntrophin (a member of the dystrophin-associated protein complex) reduces the level of AQP4 and increases hyperthermia-induced seizure susceptibility [68]. On the other hand, we implanted the infusion cannula and electrode in animals. These manipulations result in neuroinflammation and astroglial activations [69]. Thus, it is plausible that neuroinflammatory events, such as microglial- and/or astroglial activation, induced by surgeries, would also affect 67-kDa LR-dystrophin/AQP4 signaling pathway and the susceptibility to SE induction. Although we performed the comparison among animal groups applied by the same manipulations, the undesirable effects of surgeries on 67-kDa LR-mediated signaling cascades and the susceptibility to SE induction could be ruled out. Therefore, our findings suggested that 67-kDa LR neutralization might increase the susceptibility to SE induction through the BBB disruption and the reduced AQP4 expression.

In the present study, we found that the cellular specific alterations in 67-kDa LR expression might mediate the differential extent of serum extravasation in a regional-specific manner by regulating vascular permeability as well as dystrophin-AQP4 expression following SE. Although we could not explain these discrepancies in the present study, it is considered that the PC and the hippocampus are not equally affected by seizure activity during SE [40,41,69,70,71]. PC is an origin for the development of widespread limbic seizures [70,71]. In addition, projection from the PC to the hippocampus forms a loop that returns to the PC via the entorhinal cortex [70,71]. Thus, this local recurrent circuit of the PC-hippocampus-entorhinal cortex-PC [70,71] may lead to the differential effects of SE on expressions of 67-kDa LR and dystrophin-AQP4 in the hippocampus and the PC. Furthermore, the PC itself shows rapid seizure activity by repeated electrical stimulation (kindling), while the hippocampus is less sensitive [70,71]. Therefore, it is likely that the differential characteristics of the susceptibility to SE induction between the PC and the hippocampus may be relevant to the regional specific severity of vasogenic edema and the responses of 67-kDa LR expression to SE. However, the present study demonstrated that 67-kDa LR neutralization was more effective in the diminishment of the dystrophin-AQP4 complex within the PC than the total hippocampus, in spite of no difference in the amount of 67-kDa LR IgG. Therefore, it is presumable that the regional specific heterogeneous astroglial properties [3,29,30] may also affect the distinct severity of the vasogenic edema formation in response to SE and 67-kDa LR IgG infusion between the PC and the hippocampus. 

PEA15 is abundantly expressed in astrocytes, and it regulates ERK1/2 activity. The phosphorylation of PEA15 at S104 and/or S116 site increases ERK1/2 activity by abrogating PEA15-ERK1/2 interaction [23,72]. Interestingly, 67-kDa LR activation leads to PEA15 phosphorylation at S104 and S116 sites [17]. Therefore, it is plausible that 67-kDa LR neutralization would also increase ERK1/2 activity by affecting PEA15 phosphorylations. However, the present study demonstrated that blockade of 67-kDa LR could not influence PEA15 expression and its phosphorylations in the total hippocampus and the PC under physiological- and post-SE conditions. Similar to the case of ERK1/2 activation, our findings suggested that PEA-15 activity might not participate in vasogenic edema formation, and dystrophin-AQP4 expression might be induced by the blockade of 67-kDa LR.

## 4. Materials and Methods

### 4.1. Experimental Animals and Chemicals

The present study was carried out on adult male Sprague-Dawley (SD) rats (7 weeks old, *n* = 112). Animals were housed in a controlled room temperature (22 ± 2 °C), humidity (55 ± 5%), and a light-dark cycle on a 12-h on-off cycle. Food and water were available *ad libitum* throughout the experiments. All experimental protocols described below were approved by the Institutional Animal Care and Use Committee of Hallym University (Chuncheon, South Korea, Hallym 2018-2, 26th April 2018). Every effort was made to reduce the number of animals employed and to minimize the animal’s discomfort. All reagents were obtained from Sigma-Aldrich (St. Louis, MO, USA), except as noted.

### 4.2. Surgery 

Under Isoflurane anesthesia (3% induction, 1.5%–2% for surgery, and 1.5% maintenance in a 65:35 mixture of N_2_O:O_2_), animals were infused each chemical into the right lateral ventricle (1 mm posterior; 1.5 mm lateral; −3.5 mm depth to the bregma) with a brain infusion kit 1 and an Alzet 1003D osmotic pump (Alzet, Cupertino, CA, USA) for 3 days. Osmotic pump contained (1) control IgG (#ab37415, Abcam, Cambridge, UK, 50 ug/mL) + vehicle, (2) control IgG + U0126 (an ERK1/2 inhibitor, 25 μM), (3) control IgG + SP600125 (a JNK inhibitor, 10 μM), (4) anti-67-kDa LR IgG (#133645, Abcam, Cambridge, UK, 50 ug/mL) + vehicle, (5) anti-67-kDa LR IgG (#133645, Abcam, Cambridge, UK, 50 ug/mL) + U0126 (25 μM), (6) anti-67-kDa LR IgG (#133645, Abcam, Cambridge, UK, 50 ug/mL) + SP600125 (10 μM), and (7) saline. In a pilot study and our previous studies [15,53], each treatment did not show behavioral and neurological defects in normal animals. Three days after surgery (infusion), animals were used for western blot and immunohistochemistry. Some animals (*n* = 7 in each group) were simultaneously implanted with a monopolar stainless steel electrode (Plastics One, Roanoke, VA, USA) into the left dorsal hippocampus (−3.8 mm posterior; 2.0 mm lateral; −2.6 mm depth). The connecting wire and electrode socket were then inserted in an electrode pedestal (Plastics One, USA) and secured to the exposed skull with dental acrylic. Three days after surgery, rats were induced with SE by lithium chloride (LiCl)-pilocarpine.

### 4.3. SE Induction and Electroencephalogram (EEG) Analysis

Rats were pretreated with an intraperitoneal injection of LiCl (127 mg/kg, i.p.) 24 h before the induction of SE. SE was induced by administration of 30 mg/kg pilocarpine (i.p.) 20 min after atropine methylbromide (5 mg/kg i.p.) [15,65,73]. Control animals received an equal volume of normal saline instead of pilocarpine after the pretreatment with atropine methylbromide. For evaluation of the effects of 67-kDa LR neutralization on the susceptibility to SE induction, EEG signals were recorded with a DAM 80 differential amplifier (0.1–3000 Hz bandpass; World Precision Instruments, Sarasota, FL, USA). EEG activity was measured during the 2 h recording sessions from each animal. The data were digitized (400 Hz) and analyzed using LabChart Pro v7 (AD Instruments, Bella Vista, New South Wales, Australia). After baseline recording for at least 30 min, animals were given pilocarpine. Time of seizure onset was defined as the time point showing a paroxysmal depolarizing shift, which lasted more than 3 s and consisted of a rhythmic discharge between 4 and 10 Hz with an amplitude of at least two times higher than the baseline EEG. EEG activity was measured during the 2 h recording sessions from each animal. Spectrograms were also automatically calculated using a Hanning sliding window with 50% overlap. Three days after SE, when vasogenic edema peaks [3], animals were used for western blot and immunohistochemistry.

### 4.4. Western Blot

After animals were sacrificed via decapitation under urethane anesthesia (1.5 g/kg, I. P.), the hippocampus and the PC were dissected out. Hippocampal and PC tissue proteins were extracted using a lysis buffer (50 mM Tris containing 50 mM HEPES (pH 7.4), ethylene glycol tetraacetic acid (EGTA, pH 8.0), 0.2% Tergitol type NP-40, 10 mM ethylenediaminetetraacetic acid (EDTA, pH 8.0), 15 mM sodium pyrophosphate, 100 mM β-glycerophosphate, 50 mM NaF, 150 mM NaCl, 2 mM sodium orthovanadate, 1 mM phenylmethylsulfonyl fluoride (PMSF), and 1 mM dithiothreitol (DTT)) containing protease inhibitor cocktail (complete, Roche Applied Sciences, Penzberg, Bavaria, Germany) and phosphatase inhibitor cocktail (PhosSTOP^®^, Roche Applied Science, Penzberg, Bavaria, Germany). Thereafter, protein concentration was determined using a Micro BCA Protein Assay Kit (Pierce Chemical, Dallas, Texas, USA). Western blot was performed by the standard protocol. The membranes were incubated with a relatively specific primary antibody (Table 1). The ECL Kit (GE Healthcare Korea, Seoul, South Korea) was used to detect signals. The bands were detected and quantified on the ImageQuant LAS4000 system (GE Healthcare Korea, Seoul, South Korea). The β-Actin antibody was used as an internal control for quantitative analysis of relative expression levels of proteins. The ratio of phosphoprotein to total protein was described as the phosphorylation ratio.

### 4.5. Immunohistochemistry

Under urethane anesthesia (1.5 g/kg, i.p.), animals were perfused via a cannula into the left ventricle of the heart with 0.9% saline followed by 4% paraformaldehyde in 0.1 M phosphate buffer (PB, pH 7.4). After perfusion, the brains were removed and post-fixed in the same fixative overnight, subsequently cryoprotection with 30% sucrose/0.1 M PBS. Brain coronal sections of 30 μm were obtained with a cryo-microtome. Standard procedures for immunohistochemistry were used to detect serum extravasation. Briefly, free-floating sections were washed 3 times in PBS (0.1 M, pH 7.3). Next, to inactivate the endogenous peroxidase, sections were incubated in 3% H_2_O_2_ and 10% methanol in PBS (0.1 M) for 20 min at room temperature. Later, sections were incubated in biotinylated rat IgG and ABC complex (Vector, #PK-6100, Burlingame, CA, USA, diluted 1:200). Tissue sections were developed in 3,3′-diaminobenzidine in 0.1 M Tris buffer and mounted on gelatin-coated slides. Some sections were incubated with a cocktail solution containing the primary antibodies (Table 1) in PBS containing 0.3% Triton X-100 overnight at room temperature. Thereafter, sections were visualized with appropriate Cy2- and Cy3-conjugated secondary antibodies. Immunoreaction was observed using an Axio Scope microscope (Carl Zeiss Korea, Seoul, South Korea). To establish the specificity of the immunostaining, a negative control test was carried out with preimmune serum instead of the primary antibody. No immunoreactivity was observed for the negative control in any structures (Figure 2A and Figure 8A). All experimental procedures in this study were performed under the same conditions and in parallel. To quantify relative intensity, sections (10 sections per each animal) were captured using an AxioImage M2 microscope. Based on merge images (yellow, colocalization of glial fibrillary acidic protein (GFAP) or SMI-71 and 67-kDa LR), GFAP-positive astrocytes or SMI-71-positive endothelial cells (green) were selected. In turn, 67-kDa LR signals (red) were captured. pERK1/2 signals in astrocytes, neurons, and microglia were also captured by the same method. Thereafter, the mean intensity of each signal was measured by using AxioVision Rel. 4.8 software. Intensity measurements were represented as the number of 256 grayscale. The intensity of each signal was standardized by setting the threshold level (mean background intensity obtained from five image inputs). Manipulation of the images was restricted to threshold and brightness adjustments to the whole image. 

### 4.6. Statistical Analysis

Quantitative data were expressed as mean ± standard error of the mean. Data were analyzed by unpaired Student’s *t*-test. One- and two-way ANOVA were also applied with Newman–Keuls posthoc test. Comparisons of data between the PC and the hippocampus were carried out with paired Student’s *t*-test. In addition, one-way repeated measure ANOVA was applied to compare the EEG power. A value of *p* < 0.05 was considered to be statistically different.

## 5. Conclusions

To the best of our knowledge, the present data revealed, for the first time, that the cellular specific alteration in 67-kDa LR expression might affect the severity of SE-induced vasogenic edema formation in regional specific manners. In addition, 67-kDa LR inhibition might regulate dystrophin-AQP4 expression in intact astrocytes, independent of JNK and PEA15 activities (Figure 13). Thus, our findings suggested that the regulation of 67-kDa LR expression/functions might be one of the considerable factors for the medication of vasogenic edema formation and prevention of its complications.

## Figures and Tables

**Table 1:** Primary antibodies used in the present study.

Antigen.	Host	Manufacturer (Catalog Number)	Dilution Used
67-kDa LR	Rabbit Rabbit	Abcam (#ab133645) Abcam, (#ab133775)	1:1000 (WB) 1:500 (IH)
AQP4	Rabbit	Alomone labs (#AQP-004)	1:5000 (WB)
Dystrophin	Rabbit	Abcam (#ab15277)	1:5000 (WB)
ERK1/2	Rabbit	Biorbyt (Orb160960)	1:2,000 (WB) 1:100 (IH)
GFAP	Mouse	Millipore (#MAB3402)	1:5000 (IH)
Iba-1	Mouse	Abcam (#ab15690)	1:100 (IH)
JNK	Rabbit	Protein tech (10023-1-AP)	1:1000
Laminin	Rabbit	Abcam (#ab11575)	1:1000 (WB)
NeuN	Guinea pig	Millipore (#ABN90P)	1:1000 (IH)
Phospho (p)-p38 MAPK	Rabbit	Abbiotec (#251246)	1:200 (WB)
p38 MAPK	Rabbit	Cell signaling (#9212)	1:1000 (WB)
PEA15	Rabbit	Antibodies-online, (ABIN1737975)	1:500 (WB)
pERK1/2	Rabbit	Bioss (bs-3330R)	1:1000 (WB) 1:100 (IH)
pJNK	Rabbit	Millipore (#07-105)	1:1000 (WB)
pPEA15-S104	Rabbit	Antibodies-online (ABIN744683)	1:500 (WB)
pPEA15-S116	Rabbit	Antibodies-online, (ABIN744698)	1:500 (WB)
rat IgG	Goat	Vector (#PI-9400)	1:200 (IH)
SMI-71	Mouse	Covance (#SMI-71R)	1:1000 (IH)
VEGF	Rabbit	Abcam (#ab46154)	1:1000 (WB)
β-actin	Mouse	Sigma (#A5316)	1:5000 (WB)

IH: Immunohistochemistry; WB: Western blot. Abbreviations: 67-kDa LR, 67-kDa laminin receptor; AQP4, aquaporin-4; ERK1/2, extracellular signal-regulated kinase 1/2; GFAP, glial fibrillary acidic protein; Iba-1, ionized calcium binding adapter molecule-1; JNK, c-Jun N-terminal kinase; NeuN, neuronal nuclear marker; p38 MAPK, p38 mitogen-activated protein kinase; PEA15, phosphoprotein enriched in astrocytes of 15 kDa; VEGF, vascular endothelial growth factor.

## Supplementary Materials

The following are available online at https://www.mdpi.com/1422-0067/20/23/6025/s1.

## References

[1] Temkin N.R. (2003). Risk factors for posttraumatic seizures in adults. Epilepsia.

[2] Jacobs M.P., Leblanc G.G., Brooks-Kayal A., Jensen F.E., Lowenstein D.H., Noebels J.L., Spencer D.D., Swann J.W. (2009). Curing epilepsy: Progress and future directions. Epilepsy Behav..

[3] Kim J.E., Yeo S.I., Ryu H.J., Kim M.J., Kim D.S., Jo S.M., Kang T.C. (2010). Astroglial loss and edema formation in the rat piriform cortex and hippocampus following pilocarpine-induced status epilepticus. J. Comp. Neurol..

[4] Sheen S.H., Kim J.E., Ryu H.J., Yang Y., Choi K.C., Kang T.C. (2011). Decrease in dystrophin expression prior to disruption of brain-blood barrier within the rat piriform cortex following status epilepticus. Brain Res..

[5] Kim Y.J., Kim J.E., Choi H.C., Song H.K., Kang T.C. (2015). Cellular and regional specific changes in multidrug efflux transporter expression during recovery of vasogenic edema in the rat hippocampus and piriform cortex. BMB Rep..

[6] Rigau V., Morin M., Rousset M.C., de Bock F., Lebrun A., Coubes P., Picot M.C., Baldy-Moulinier M., Bockaert J., Crespel A. (2007). Angiogenesis is associated with blood-brain barrier permeability in temporal lobe epilepsy. Brain.

[7] Thyboll J., Kortesmaa J., Cao R., Soininen R., Wang L., Iivanainen A., Sorokin L., Risling M., Cao Y., Tryggvason K. (2002). Deletion of the laminin alpha4 chain leads to impaired microvessel maturation. Mol. Cell. Biol..

[8] Miner J.H. (2008). Laminins and their roles in mammals. Microsc. Res. Tech..

[9] Lesot H., Kuhl U., Mark K. (1983). Isolation of a laminin-binding protein from muscle cell membranes. EMBO J..

[10] Rao C.N., Castronovo V., Schmitt M.C., Wewer U.M., Claysmith A.P., Liotta L.A., Sobel M.E. (1989). Evidence for a precursor of the high-affinity metastasis-associated murine laminin receptor. Biochemistry.

[11] McCaffery P., Neve R.L., Drager U.C. (1990). A dorso-ventral asymmetry in the embryonic retina defined by protein conformation. Proc. Natl. Acad. Sci. USA.

[12] Tohgo A., Takasawa S., Munakata H., Yonekura H., Hayashi N., Okamoto H. (1994). Structural determination and characterization of a 40 kDa protein isolated from rat 40 S ribosomal subunit. FEBS Lett..

[13] Pellegrini R., Martignone S., Menard S., Colnaghi M.I. (1994). Laminin receptor expression and function in small-cell lung carcinoma. Int. J. Cancer.

[14] Ardini E., Sporchia B., Pollegioni L., Modugno M., Ghirelli C., Castiglioni F., Tagliabue E., Ménard S. (2002). Identification of a novel function for 67-kDa laminin receptor: Increase in laminin degradation rate and release of motility fragments. Cancer Res..

[15] Park H., Choi S.H., Kong M.J., Kang T.C. (2019). Dysfunction of 67-kDa laminin receptor disrupts BBB integrity via impaired dystrophin/AQP4 complex and p38 MAPK/VEGF activation following status epilepticus. Front. Cell. Neurosci..

[16] Givant-Horwitz V., Davidson B., Reich R. (2004). Laminin-induced signaling in tumor cells: The role of the M(r) 67,000 laminin receptor. Cancer Res..

[17] Formisano P., Ragno P., Pesapane A., Alfano D., Alberobello A.T., Rea V.E., Giusto R., Rossi F.W., Beguinot F., Rossi G. (2012). PED/PEA-15 interacts with the 67 kD laminin receptor and regulates cell adhesion, migration, proliferation and apoptosis. J. Cell. Mol. Med..

[18] Fiory F., Formisano P., Perruolo G., Beguinot F. (2009). Frontiers: PED/PEA-15, a multifunctional protein controlling cell survival and glucose metabolism. Am. J. Physiol. Endocrinol. Metab..

[19] Araujo H., Danziger N., Cordier J., Glowinski J., Chneiweiss H. (1993). Characterization of PEA-15, a major substrate for protein kinase C in astrocytes. J. Biol. Chem..

[20] Estellés A., Yokoyama M., Nothias F., Vincent J.D., Glowinski J., Vernier P., Chneiweiss H. (1996). The major astrocytic phosphoprotein PEA-15 is encoded by two mRNAs conserved on their full length in mouse and human. J. Biol. Chem..

[21] Kubes M., Cordier J., Glowinski J., Girault J.A., Chneiweiss H. (1998). Endothelin induces a calcium-dependent phosphorylation of PEA-15 in intact astrocytes: Identification of Ser104 and Ser116 phosphorylated, respectively, by protein kinase C and calcium/calmodulin kinase II in vitro. J. Neurochem..

[22] Trencia A., Perfetti A., Cassese A., Vigliotta G., Miele C., Oriente F., Santopietro S., Giacco F., Condorelli G., Formisano P. (2003). Protein kinase B/Akt binds and phosphorylates PED/PEA-15, stabilizing its antiapoptotic action. Mol. Cell. Biol..

[23] Krueger J., Chou F.L., Glading A., Schaefer E., Ginsberg M.H. (2005). Phosphorylation of phosphoprotein enriched in astrocytes (PEA-15) regulates extracellular signal-regulated kinase-dependent transcription and cell proliferation. Mol. Biol. Cell..

[24] Baloui H., von Boxberg Y., Vinh J., Weiss S., Rossier J., Nothias F., Stettler O. (2004). Cellular prion protein/laminin receptor: Distribution in adult central nervous system and characterization of an isoform associated with a subtype of cortical neurons. Eur. J. Neurosci..

[25] Jovanovic K., Chetty C.J., Khumalo T., Da Costa Dias B., Ferreira E., Malindisa S.T., Caveney R., Letsolo B.T., Weiss S.F. (2015). Novel patented therapeutic approaches targeting the 37/67 kDa laminin receptor for treatment of cancer and Alzheimer’s disease. Expert Opin. Ther. Pat..

[26] Neely J.D., Amiry-Moghaddam M., Ottersen O.P., Froehner S.C., Agre P., Adams M.E. (2001). Syntrophin-dependent expression and localization of Aquaporin-4 water channel protein. Proc. Natl. Acad. Sci. USA.

[27] Nico B., Frigeri A., Nicchia G.P., Corsi P., Ribatti D., Quondamatteo F., Herken R., Girolamo F., Marzullo A., Svelto M. (2003). Severe alterations of endothelial and glial cells in the blood-brain barrier of dystrophic mdx mice. Glia.

[28] Wolburg H., Noell S., Mack A., Wolburg-Buchholz K., Fallier-Becker P. (2009). Brain endothelial cells and the glio-vascular complex. Cell Tissue Res..

[29] Kim D.S., Kim J.E., Kwak S.E., Choi K.C., Kim D.W., Kwon O.S., Choi S.Y., Kang T.C. (2008). Spatiotemporal characteristics of astroglial death in the rat hippocampo-entorhinal complex following pilocarpine-induced status epilepticus. J. Comp. Neurol..

[30] Kim J.E., Kim Y.J., Kim J.Y., Kang T.C. (2014). PARP1 activation/expression modulates regional-specific neuronal and glial responses to seizure in a hemodynamic-independent manner. Cell Death Dis..

[31] Seiffert E., Dreier J.P., Ivens S., Bechmann I., Tomkins O., Heinemann U., Friedman A. (2004). Lasting blood-brain barrier disruption induces epileptic focus in the rat somatosensory cortex. J. Neurosci..

[32] Papadopoulos M.C., Manley G.T., Krishna S., Verkman A.S. (2004). Aquaporin-4 facilitates reabsorption of excess fluid in vasogenic brain edema. FASEB J..

[33] Bloch O., Papadopoulos M.C., Manley G.T., Verkman A.S. (2005). Aquaporin-4 gene deletion in mice increases focal edema associated with staphylococcal brain abscess. J. Neurochem..

[34] Warth A., Kröger S., Wolburg H. (2004). Redistribution of aquaporin-4 in human glioblastoma correlates with loss of agrin immunoreactivity from brain capillary basal laminae. Acta Neuropathol..

[35] Enger R., Gundersen G.A., Haj-Yasein N.N., Eilert-Olsen M., Thoren A.E., Vindedal G.F., Petersen P.H., Skare Ø., Nedergaard M., Ottersen O.P. (2012). Molecular scaffolds underpinning macroglial polarization: An analysis of retinal Müller cells and brain astrocytes in mouse. Glia.

[36] El Mathari B., Sene A., Charles-Messance H., Vacca O., Guillonneau X., Grepin C., Sennlaub F., Sahel J.A., Rendon A., Tadayoni R. (2015). Dystrophin Dp71 gene deletion induces retinal vascular inflammation and capillary degeneration. Hum. Mol. Genet..

[37] Vacca O., Charles-Messance H., El Mathari B., Sene A., Barbe P., Fouquet S., Aragón J., Darche M., Giocanti-Aurégan A., Paques M. (2016). AAV-mediated gene therapy in Dystrophin-Dp71 deficient mouse leads to blood-retinal barrier restoration and oedema reabsorption. Hum. Mol. Genet..

[38] Wolburg H., Noell S., Fallier-Becker P., Mack A.F., Wolburg-Buchholz K. (2012). The disturbed blood-brain barrier in human glioblastoma. Mol. Aspects Med..

[39] Turski L., Cavalheiro E.A., Turski W.A., Meldrum B.S. (1986). Excitatory neurotransmission within substantia nigra pars reticulata regulates threshold for seizures produced by pilocarpine in rats: Effects of intranigral 2-amino-7-phosphonoheptanoate and N-methyl-D-aspartate. Neuroscience.

[40] Turski W.A., Cavalheiro E.A., Schwarz M., Czuczwar S.J., Kleinrok Z., Turski L. (1983). Limbic seizures produced by pilocarpine in rats: A behavioural, electroencephalographic and neuropathological study. Behav. Brain Res..

[41] Fujikawa D.G. (1996). The temporal evolution of neuronal damage from pilocarpine-induced status epilepticus. Brain Res..

[42] Kang T.C., Kim D.S., Kwak S.E., Kim J.E., Won M.H., Kim D.W., Choi S.Y., Kwon O.S. (2006). Epileptogenic roles of astroglial death and regeneration in the dentate gyrus of experimental temporal lobe epilepsy. Glia.

[43] Schmidt-Kastner R., Ingvar M. (1994). Loss of immunoreactivity for glial fibrillary acidic protein (GFAP) in astrocytes as a marker for profound tissue damage in substantia nigra and basal cortical areas after status epilepticus induced by pilocarpine in rat. Glia.

[44] Schmidt-Kastner R., Ingvar M. (1996). Laminar damage of neurons and astrocytes in neocortex and hippocampus of rat after long-lasting status epilepticus induced by pilocarpine. Epilepsy Res. Suppl..

[45] Tachibana H., Koga K., Fujimura Y., Yamada K. (2004). A receptor for green tea polyphenol EGCG. Nat. Struct. Mol. Biol..

[46] Byun E.B., Choi H.G., Sung N.Y., Byun E.H. (2012). Green tea polyphenol epigallocatechin-3-gallate inhibits TLR4 signaling through the 67-kDa laminin receptor on lipopolysaccharide-stimulated dendritic cells. Biochem. Biophys. Res. Commun..

[47] Wang Q.M., Wang H., Li Y.F., Xie Z.Y., Ma Y., Yan J.J., Gao Y.F., Wang Z.M., Wang L.S. (2016). Inhibition of EMMPRIN and MMP-9 Expression by Epigallocatechin-3-Gallate through 67-kDa Laminin Receptor in PMA-Induced Macrophages. Cell Physiol. Biochem..

[48] Ku H.C., Chang H.H., Liu H.C., Hsiao C.H., Lee M.J., Hu Y.J., Hung P.F., Liu C.W., Kao Y.H. (2009). Green tea (-)-epigallocatechin gallate inhibits insulin stimulation of 3T3-L1 preadipocyte mitogenesis via the 67-kDa laminin receptor pathway. Am. J. Physiol. Cell. Physiol..

[49] Ku H.C., Liu H.S., Hung P.F., Chen C.L., Liu H.C., Chang H.H., Tsuei Y.W., Shih L.J., Lin C.L., Lin C.M. (2012). Green tea (-)-epigallocatechin gallate inhibits IGF-I and IGF-II stimulation of 3T3-L1 preadipocyte mitogenesis via the 67-kDa laminin receptor, but not AMP-activated protein kinase pathway. Mol. Nutr. Food Res..

[50] Shi Z.F., Zhao W.J., Xu L.X., Dong L.P., Yang S.H., Yuan F. (2015). Downregulation of aquaporin 4 expression through extracellular signal-regulated kinases1/2 activation in cultured astrocytes following scratch-injury. Biomed. Env. Sci..

[51] Qi L.L., Fang S.H., Shi W.Z., Huang X.Q., Zhang X.Y., Lu Y.B., Zhang W.P., Wei E.Q. (2011). CysLT2 receptor-mediated AQP4 up-regulation is involved in ischemic-like injury through activation of ERK and p38 MAPK in rat astrocytes. Life Sci..

[52] Salman M.M., Sheilabi M.A., Bhattacharyya D., Kitchen P., Conner A.C., Bill R.M., Woodroofe M.N., Conner M.T., Princivalle A.P. (2017). Transcriptome analysis suggests a role for the differential expression of cerebral aquaporins and the MAPK signalling pathway in human temporal lobe epilepsy. Eur. J. Neurosci..

[53] Ko A.R., Kang T.C. (2017). TRPC6-mediated ERK1/2 phosphorylation prevents dentate granule cell degeneration via inhibiting mitochondrial elongation. Neuropharmacology.

[54] Kim J.E., Kang T.C. (2018). Nucleocytoplasmic p27(Kip1) export is required for ERK1/2-mediated reactive astroglial proliferation following status epilepticus. Front. Cell. Neurosci..

[55] Min S.J., Hyun H.W., Kang T.C. (2017). Leptomycin B attenuates neuronal death via PKA- and PP2B-mediated ERK1/2 activation in the rat hippocampus following status epilepticus. Brain Res..

[56] Liu L., Sun L., Zhao P., Yao L., Jin H., Liang S., Wang Y., Zhang D., Pang Y., Shi Y. (2010). Hypoxia promotes metastasis in human gastric cancer by up-regulating the 67-kDa laminin receptor. Cancer Sci..

[57] Zhang G., Ma P., Wan S., Xu J., Yang M., Qiu G., Zhuo F., Xu S., Huo J., Ju Y. (2019). Dystroglycan is involved in the activation of ERK pathway inducing the change of AQP4 expression in scratch-injured astrocytes. Brain Res..

[58] Leucht C., Simoneau S., Rey C., Vana K., Rieger R., Lasmézas C.I., Weiss S. (2003). The 37 kDa/67 kDa laminin receptor is required for PrP(Sc) propagation in scrapie-infected neuronal cells. EMBO Rep..

[59] Salama R.H., Muramatsu H., Zou K., Inui T., Kimura T., Muramatsu T. (2001). Midkine binds to 37-kDa laminin binding protein precursor, leading to nuclear transport of the complex. Exp. Cell Res..

[60] Chung J.W., Hong S.J., Kim K.J., Goti D., Stins M.F., Shin S., Dawson V.L., Dawson T.M., Kim K.S. (2003). 37-kDa laminin receptor precursor modulates cytotoxic necrotizing factor 1-mediated RhoA activation and bacterial uptake. J. Biol. Chem..

[61] Kim J.E., Kang T.C. (2017). p47Phox/CDK5/DRP1-mediated mitochondrial fission evokes PV cell degeneration in the rat dentate gyrus following status epilepticus. Front. Cell. Neurosci..

[62] Kim J.E., Kang T.C. (2018). Differential roles of mitochondrial translocation of active caspase-3 and HMGB1 in neuronal death induced by status epilepticus. Front. Cell. Neurosci..

[63] Gundimeda U., McNeill T.H., Barseghian B.A., Tzeng W.S., Rayudu D.V., Cadenas E., Gopalakrishna R. (2015). Polyphenols from green tea prevent antineuritogenic action of Nogo-A via 67-kDa laminin receptor and hydrogen peroxide. J. Neurochem..

[64] Uva L., Librizzi L., Marchi N., Noe F., Bongiovanni R., Vezzani A., Janigro D., de Curtis M. (2008). Acute induction of epileptiform discharges by pilocarpine in the in vitro isolated guinea-pig brain requires enhancement of blood-brain barrier permeability. Neuroscience.

[65] Roch C., Leroy C., Nehlig A., Namer I.J. (2002). Magnetic resonance imaging in the study of the lithium-pilocarpine model of temporal lobe epilepsy in adult rats. Epilepsia.

[66] Binder D.K., Yao X., Zador Z., Sick T.J., Verkman A.S., Manley G.T. (2006). Increased seizure duration and slowed potassium kinetics in mice lacking aquaporin-4 water channels. Glia.

[67] Lee D.J., Hsu M.S., Seldin M.M., Arellano J.L., Binder D.K. (2012). Decreased expression of the glial water channel aquaporin-4 in the intrahippocampal kainic acid model of epileptogenesis. Exp. Neurol..

[68] Amiry-Moghaddam M., Williamson A., Palomba M., Eid T., de Lanerolle N.C., Nagelhus E.A., Adams M.E., Froehner S.C., Agre P., Ottersen O.P. (2003). Delayed K+ clearance associated with aquaporin-4 mislocalization: Phenotypic defects in brains of alpha-syntrophin-null mice. Proc. Natl. Acad. Sci. USA..

[69] Salatino J.W., Ludwig K.A., Kozai T.D.Y., Purcell E.K. (2017). Glial responses to implanted electrodes in the brain. Nat. Biomed. Eng..

[70] Ramsdell J.S., Gulland F.M. (2014). Domoic acid epileptic disease. Mar. Drugs.

[71] Vaughan D.N., Jackson G.D. (2014). The piriform cortex and human focal epilepsy. Front. Neurol..

[72] Danziger N., Yokoyama M., Jay T., Cordier J., Glowinski J., Chneiweiss H. (1995). Cellular expression, developmental regulation, and phylogenic conservation of PEA-15, the astrocytic major phosphoprotein and protein kinase C substrate. J. Neurochem..

[73] Dubé C., André V., Covolan L., Ferrandon A., Marescaux C., Nehlig A. (1998). C-Fos, Jun D and HSP72 immunoreactivity, and neuronal injury following lithium-pilocarpine induced status epilepticus in immature and adult rats. Brain Res. Mol. Brain Res..

