# Supplementary material for: The Regional Specific Alterations in BBB Permeability are Relevant to the Differential Responses of 67-kDa LR Expression in Endothelial Cells and Astrocytes Following Status Epilepticus
Source: Int J Mol Sci. 2019 Nov 29;20(23):6025. doi: 10.3390/ijms20236025 (PMC6929072; doi:10.3390/ijms20236025)
Supplement: Supplementary file 1 [file ijms-20-06025-s001.pdf]

Supplementary information

The regional specific alterations in BBB permeability are relevant to the differential responses of 67-kDa LR expression in endothelial cells and astrocytes following status epilepticus

Hana Park[1,2], Tae-Cheon Kang[1,2*]

[1]Department of Anatomy and Neurobiology, College of Medicine, Hallym University, Chuncheon 24252, South Korea

[2]Institute of Epilepsy Research, College of Medicine, Hallym University, Chuncheon 24252, South Korea

Running title: 67-kDa LR-mediated regional specific vasogenic edema

* Correspondence to: T. -C. Kang, Department of Anatomy and Neurobiology, College of Medicine, Hallym University, Chuncheon, Kangwon-Do 24252, South Korea; Tel: +82-33-248-2524; Fax: +82-33-248-2525; E-mail: tckang@hallym.ac.kr

Fig. 1C

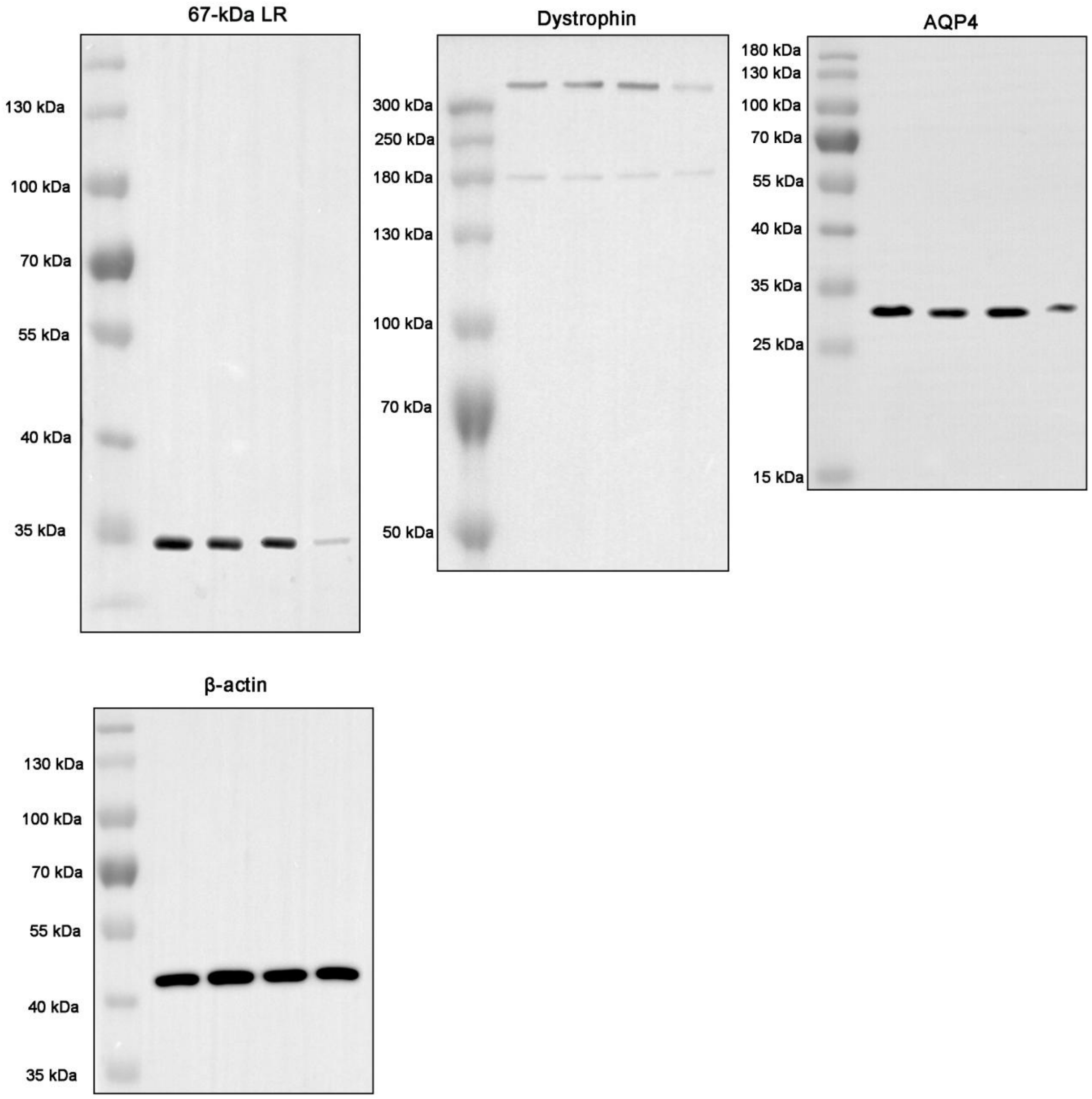

Supplementary Fig. 1. Full-length gel images of western blot data in Fig. 1C.

Fig. 4A

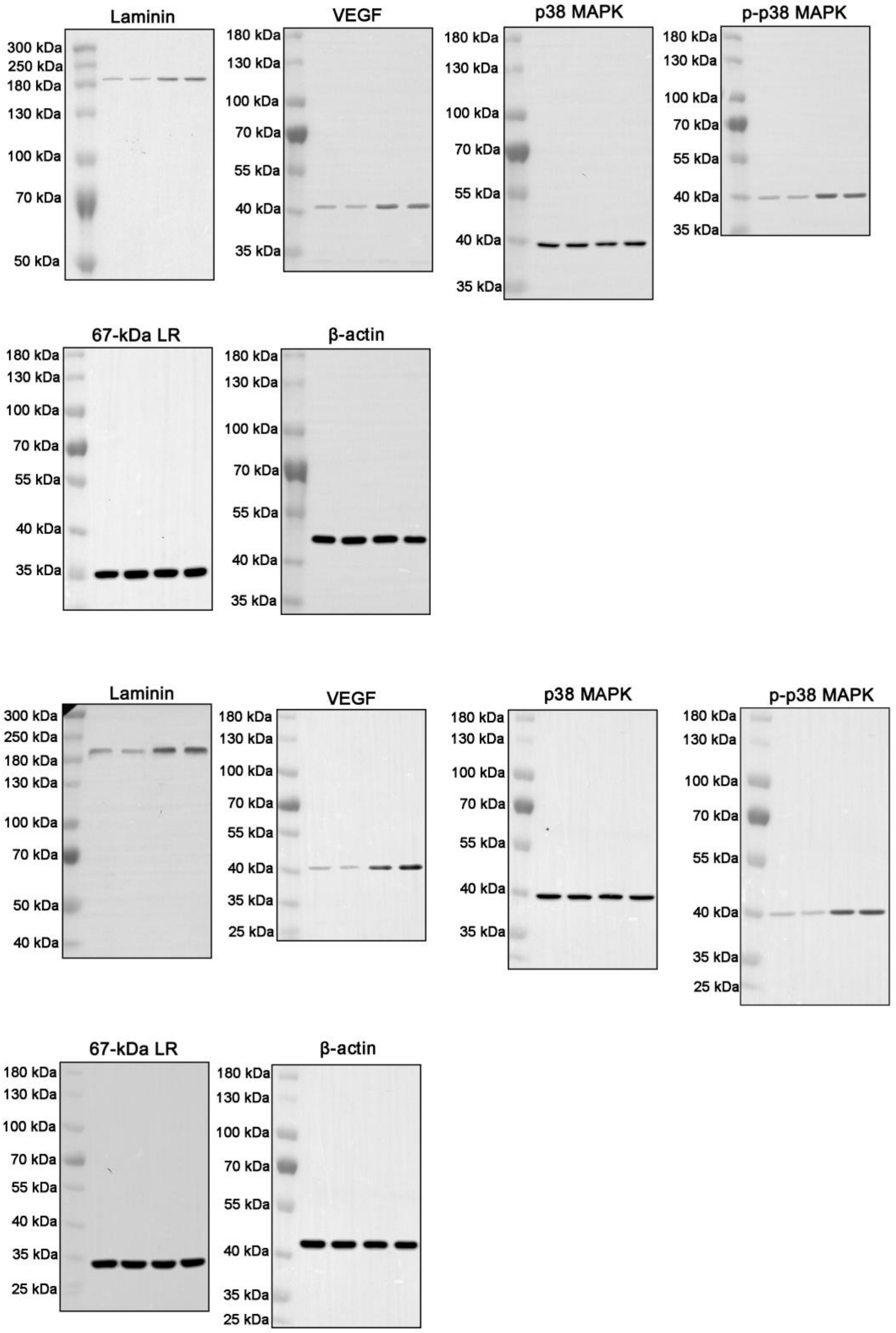

Supplementary Fig. 2. Full-length gel images of western blot data in Fig. 4A.

Fig. 6A

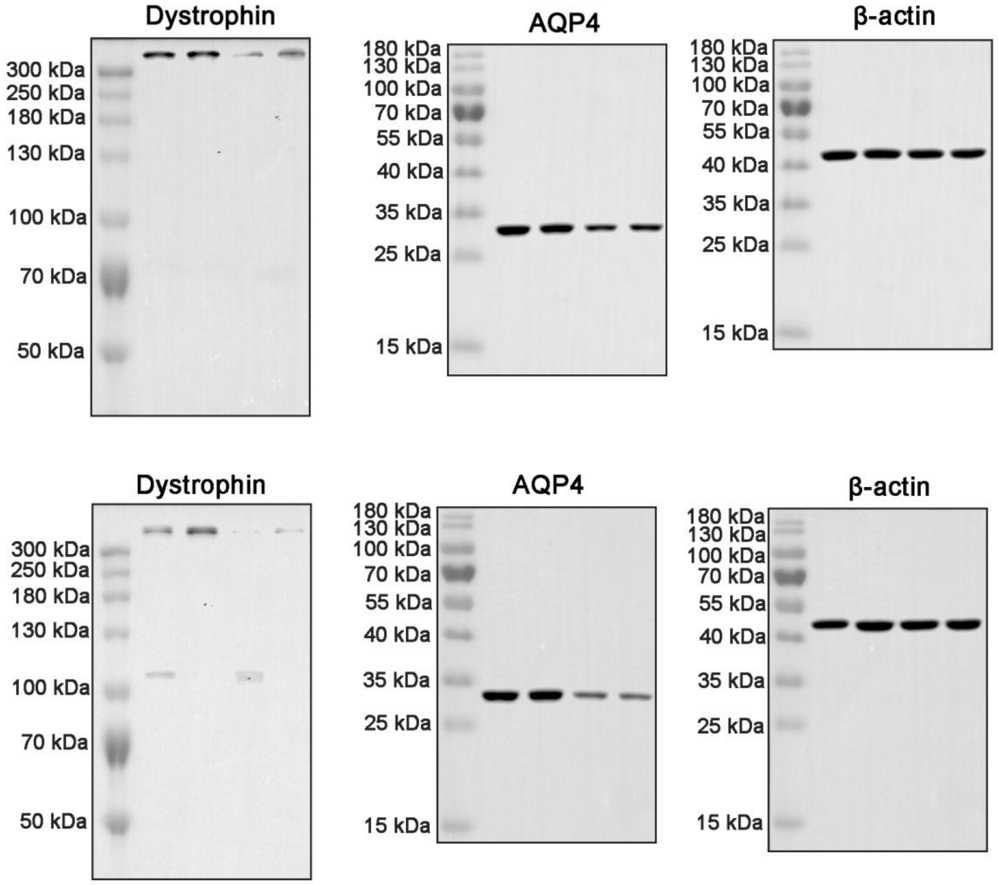

Fig. 6C

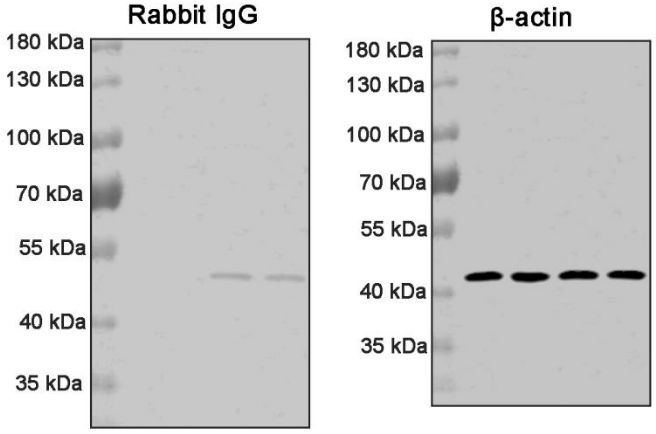

Supplementary Fig. 3. Full-length gel images of western blot data in Figs. 6A and 6C.

Fig. 7A

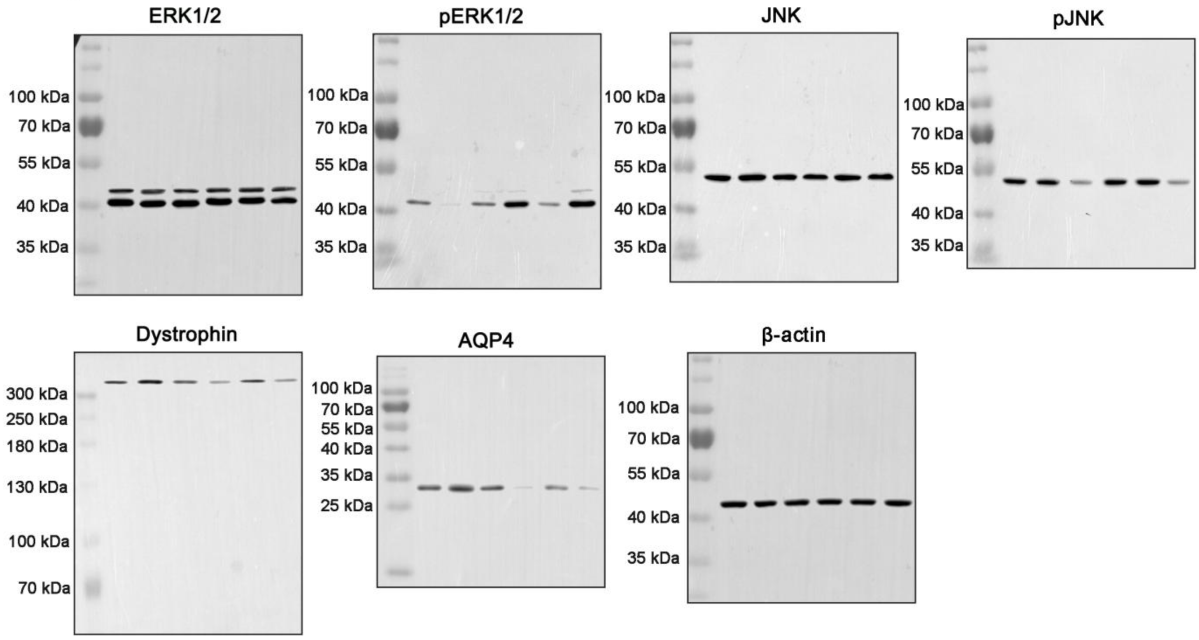

Fig. 9A

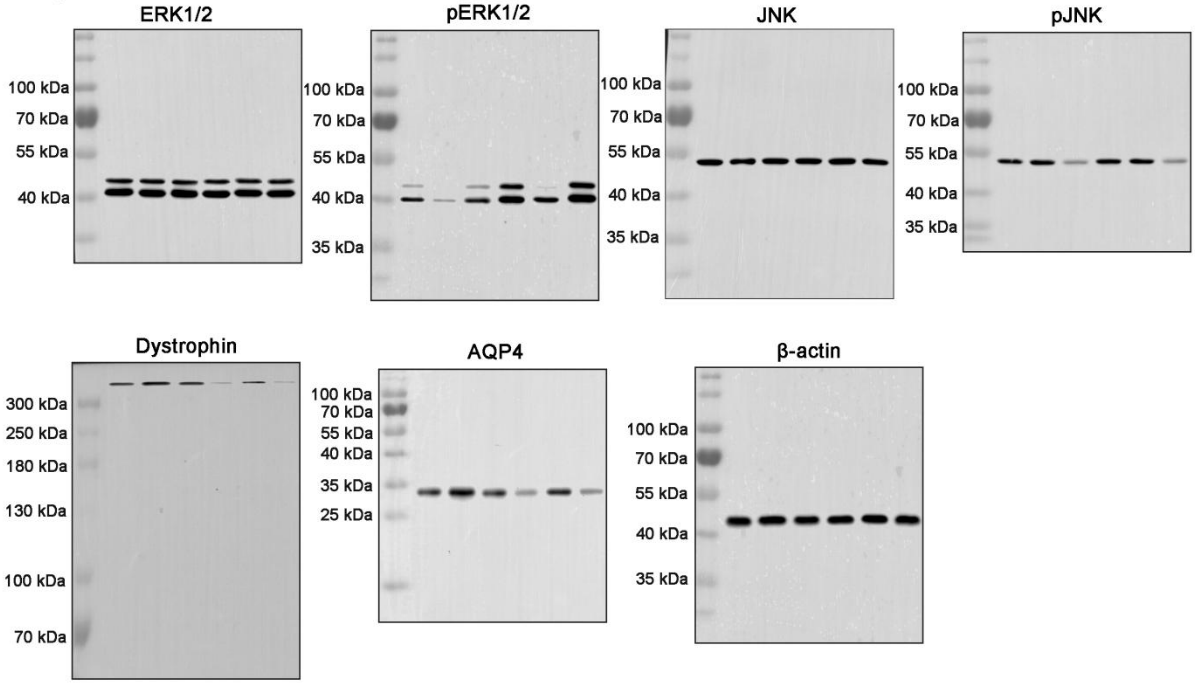

Supplementary Fig. 4. Full-length gel images of western blot data in Figs. 7A and 9A.

Fig. 11A

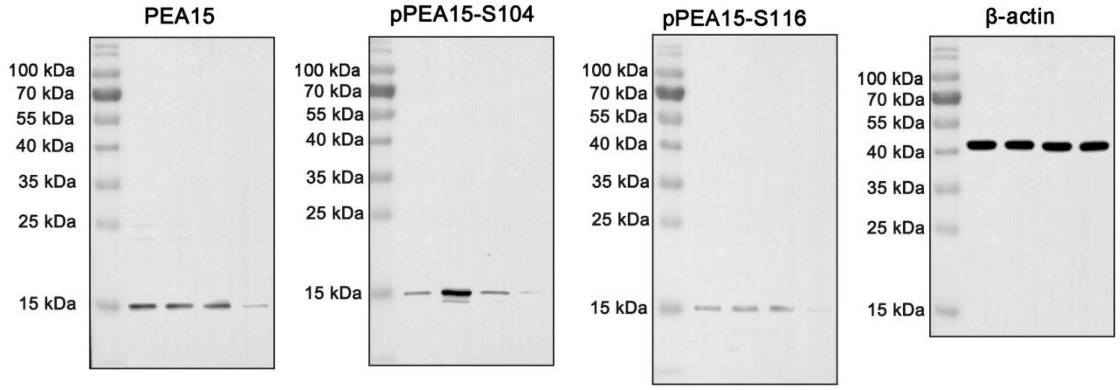

Fig. 11C

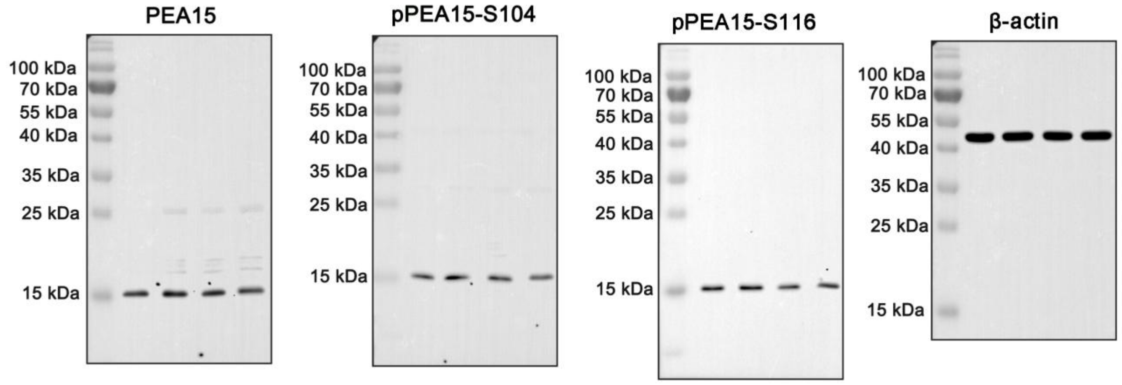

Supplementary Fig. 5. Full-length gel images of western blot data in Fig. 11A and 11C.
